# Clinical Impact of Intraoperative Margin Assessment in Breast-Conserving Surgery With a Novel Pegulicianine Fluorescence–Guided System: A Nonrandomized Controlled Trial

**DOI:** 10.1001/jamasurg.2022.1075

**Published:** 2022-05-11

**Authors:** E. Shelley Hwang, Peter Beitsch, Peter Blumencranz, David Carr, Anees Chagpar, Lynne Clark, Nayana Dekhne, Daleela Dodge, Donna L. Dyess, Linsey Gold, Stephen Grobmyer, Kelly Hunt, Stephen Karp, Beth-Ann Lesnikoski, Irene Wapnir, Barbara L. Smith

**Affiliations:** 1 Duke Cancer Institute and Duke University Health System, Durham, North Carolina; 2 Dallas Surgical Group, Dallas, Texas; 3 The Comprehensive Breast Care Center, BayCare Medical Group, Clearwater, Florida; 4 Novant Health, Winston-Salem, North Carolina; 5 Yale-New Haven Hospital, New Haven, Connecticut; 6 CHI Franciscan, Tacoma, Washington; 7 Beaumont Hospital, Royal Oak, Michigan; 8 Penn State Health, Hershey, Pennsylvania; 9 Mitchell Cancer Institute, University of South Alabama, Mobile; 10 Beaumont Hospital, Troy, Michigan; 11 Cleveland Clinic Foundation, Cleveland, Ohio; 12 MD Anderson Cancer Center, Houston, Texas; 13 Beth Israel Lahey Health, Burlington, Massachusetts; 14 Baptist MD Anderson, Jacksonville, Florida; 15 Stanford Healthcare, Palo Alto, California; 16 Massachusetts General Hospital, Boston

## Abstract

**Importance:**

Positive margins following breast-conserving surgery (BCS) are often identified on standard pathology evaluation. Intraoperative assessment of the lumpectomy cavity has the potential to reduce residual disease or reexcision rate following standard of care BCS in real time.

**Objective:**

To collect safety and initial efficacy data on the novel pegulicianine fluorescence–guided system (pFGS) when used to identify residual cancer in the tumor bed of female patients undergoing BCS.

**Design, Setting, and Participants:**

This prospective single-arm open-label study was conducted as a nonrandomized multicenter controlled trial at 16 academic or community breast centers across the US. Female patients 18 years and older with newly diagnosed primary invasive breast cancer or ductal carcinoma in situ DCIS undergoing BCS were included, excluding those with previous breast cancer surgery and a history of dye allergies. Of 283 consecutive eligible patients recruited, 234 received a pegulicianine injection and were included in the safety analysis; of these, 230 were included in the efficacy analysis. Patients were enrolled between February 6, 2018, and April 10, 2020, and monitored for a 30-day follow-up period. Data were analyzed from April 10, 2020, to August 5, 2021.

**Interventions:**

Participants received an injection of a novel imaging agent (pegulicianine) a mean (SD) of 3.2 (0.9) hours prior to surgery at a dose of 1 mg/kg. After completing standard of care (SOC) excision, pFGS was used to scan the lumpectomy cavity to guide the removal of additional shave margins.

**Main Outcomes and Measures:**

Adverse events and sensitivity, specificity, and reexcision rate.

**Results:**

Of 234 female patients enrolled (median [IQR] age, 62.0 [55.0-69.0] years), 230 completed the trial and 1 patient with a history of allergy to contrast agents had an anaphylactic reaction and recovered without sequelae. Correlation of pFGS with final margin status on a per-margin analysis showed a marked improvement in sensitivity over standard pathology assessment of the main lumpectomy specimen (69.4% vs 38.2%, respectively). On a per-patient level, the false-negative rate of pFGS was 23.7% (9 of 38), and sensitivity was 76.3% (29 of 38). Among 32 patients who underwent excision of pFGS-guided shaves, pFGS averted the need for reexcision in 6 (19%).

**Conclusions and Relevance:**

In this pilot feasibility study, the safety profile of pegulicianine was consistent with other imaging agents used in BCS, and was associated with a reduced need for second surgery in patients who underwent intraoperative additional excision of pFGS-guided shaves. These findings support further development and clinical performance assessment of pFGS in a prospective randomized trial.

**Trial Registration:**

ClinicalTrials.gov Identifier: NCT03321929

## Key Points

**Question:**

Is the novel pegulicianine fluorescence–guided system (pFGS) safe and effective in improving outcome for patients undergoing breast-conserving surgery?

**Findings:**

In this nonrandomized controlled trial including 230 patients, 1 patient with a history of allergy to contrast agents had an anaphylactic reaction and recovered without sequelae. pFGS showed a high sensitivity rate for residual tumor, and averted the need for reexcision by 19% among patients who underwent excision of pFGS-guided shaves.

**Meaning:**

pFGS had an excellent safety profile and reduced the reexcision rate for patients undergoing breast-conserving surgery.

## Teaser

This nonrandomized controlled trial evaluates the safety and efficacy of the novel pegulicianine fluorescence–guided system in identifying residual cancer in the tumor bed of female patients undergoing breast-conserving surgery.

## Introduction

Preventing in-breast recurrence is a primary goal for breast-conserving surgery (BCS), as local recurrence can lead to additional surgery, further systemic therapy, and poorer cosmetic outcomes. Prevention of local recurrence may also lead to improved overall survival, as suggested by a meta-analysis^1^ of randomized clinical trials that showed 1 excess death for every 4 ipsilateral breast tumor recurrences. The strongest current predictor of local recurrence is the presence of tumor at the lumpectomy margins.^2,3,4,5,6^ The site of local recurrence is often close to the original tumor site with histological characteristics similar to the primary tumor, suggesting that local recurrences can arise from residual tumor left at the time of lumpectomy.^7,8,9^ Rates of residual carcinoma in reexcisions or mastectomies following initially negative lumpectomy margins have been shown to exceed 40% in some cohorts.^10,11,12,13^

Preoperative imaging and current intraoperative examination methods do not accurately identify the microscopic extent of tumors, making it challenging to achieve complete tumor excision during BCS. As a result, positive margins determined several days after surgery by pathology examination require reexcision after initial lumpectomy in 20% to 40% of patients.^14,15,16,17,18^ An ideal approach to reduce reexcision rates would include comprehensive intraoperative evaluation of the lumpectomy cavity, identification and removal of residual tumor, and verification that negative margins have been achieved, all during the initial surgery. Such a technique may also allow surgeons to excise the tumor with narrower margins by removing additional tissue only at sites of residual tumor.

To address this challenge, Lumicell has developed a novel fluorescence-guided system (FGS) comprising an activatable fluorescent imaging agent (pegulicianine),^19^ a handheld device,^20^ and a patient-specific tumor detection algorithm.^21^ Pegulicianine FGS (pFGS) had been developed in an earlier single-center study in 55 patients^20,21^ for identifying residual tumor in real time through direct interrogation of the lumpectomy cavity. The findings suggested that pFGS had potential to address the current limitations of standard of care (SOC) BCS. This study reports the results of a multicenter feasibility study that further evaluated the performance and safety of pFGS when used to identify residual cancer in the tumor bed of female breast cancer patients undergoing BCS.

## Methods

### Patient Selection

The Western Institutional Review Board, Puyallup, Washington, approved this study, and written informed consent was obtained from all participants. The study followed the Consolidated Standards of Reporting Trials (CONSORT) reporting guideline. Patients were recruited at 16 US academic and community breast centers (eTable 1 in Supplement 3). Eligible participants included female patients 18 years and older with invasive breast cancer and/or ductal carcinoma in situ (DCIS). Patients treated with neoadjuvant therapy, undergoing margin reexcision following prior BCS, or injected with blue dyes for sentinel node mapping before completing the pFGS procedure were excluded from the study. Based on our earlier study,^21^ we anticipated that up to 5 patients per surgeon and approximately 1 to 3 surgeons per site (up to 250 patients total) would be required to address any site-specific and surgeon-specific usability issues related to the implementation of pFGS.

### pFGS Procedures

**Figure 1.**
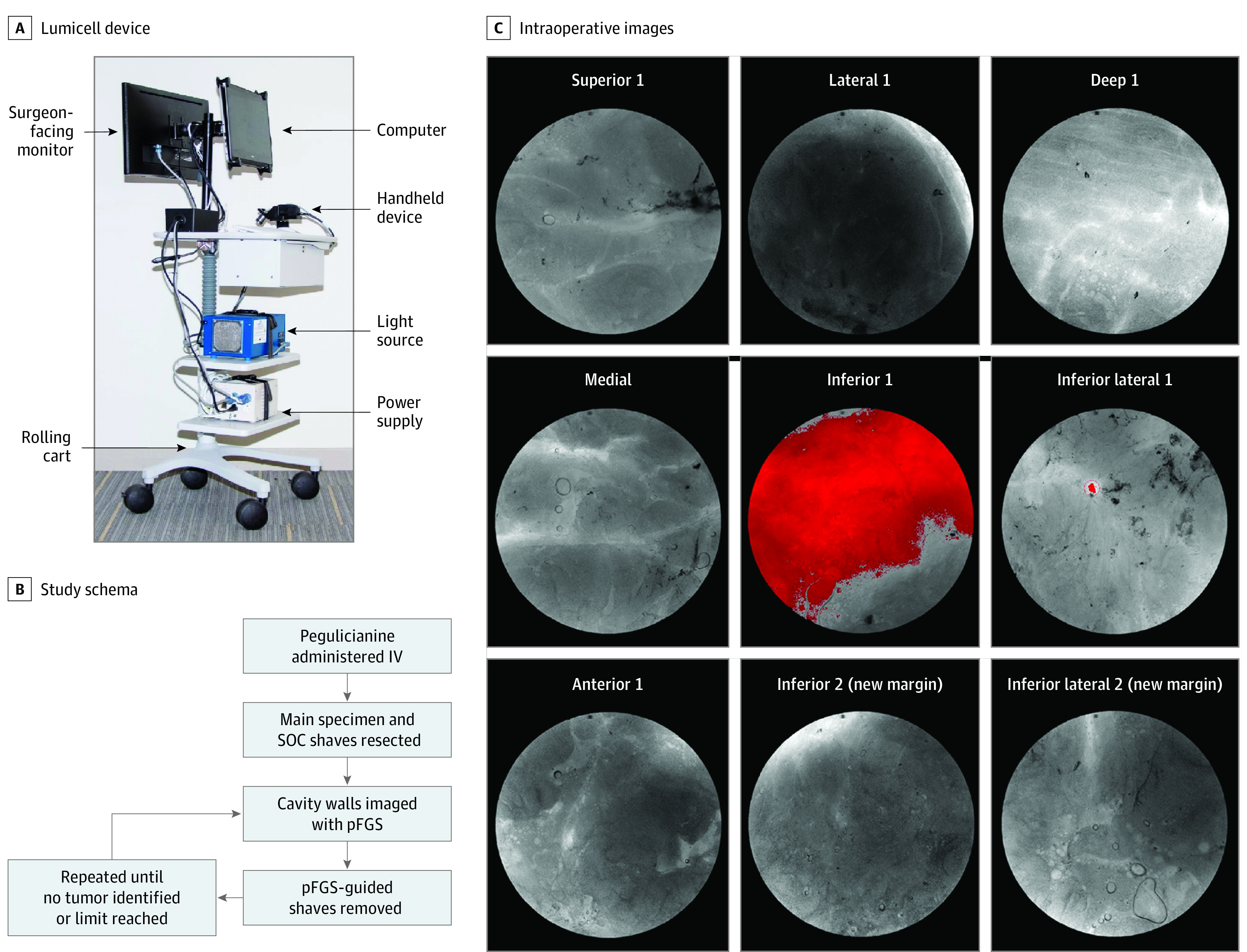
Pegulicianine Fluorescence–Guided System (pFGS) Tumor Detection Device and Protocol A, The Lumicell device was installed at each site with all components mounted on a mobile tower. On-site hands-on training was conducted for the surgeons, operating room staff, and study team prior to study activation. A, The Lumicell study monitor was present either in person or virtually for each procedure to oversee device use and data collection. B, Study schema for intraoperative use of the Lumicell device. Up to 2 additional shave margins were allowed per margin orientation. C, Intraoperative images demonstrating a red signal indicating pFGS uptake. Additional shave margins were excised in the inferior and inferior lateral orientations with resultant elimination of signal. IV indicates intravenously.

Study participants were administered 1 mg/kg of pegulicianine intravenously over 3 minutes on the day of surgery. Patients were monitored for adverse events for 2 to 6 hours after infusion, then underwent SOC BCS. SOC BCS was defined as resection of the main tumor specimen as well as any shaved cavity margins deemed necessary by the surgeon (SOC shaves) to obtain grossly negative margins. After the SOC BCS was completed, imaging of the lumpectomy cavity was performed with the pFGS device. If the tumor detection algorithm identified a region suspected of containing residual tumor, the protocol required the surgeon to remove additional shaves from that region (Figure 1). The number of pFGS-guided shaves was initially limited to 1 per orientation and was later increased to 2 per orientation based on findings after the initial 127 patients.

### Safety Assessment

All participants were observed for any potential adverse reactions to the injection of pegulicianine or use of pFGS from the time of injection until hospital discharge. Participants had a final safety assessment at the first postoperative visit. Any reported adverse events were monitored until resolution. All adverse event data and data analyses were reviewed by the study’s independent data safety monitoring board.

### Final Margin Assessment

Final margin status was determined for each orientation using standard histopathology examination of the outermost surface of the resected specimens (main specimen, SOC shave, or pFGS-guided shave). Positive margins were defined according to the Society of Surgical Oncology consensus guidelines^2,3^ as ink on tumor for invasive cancer with or without DCIS component or cancer cells present within 2 mm from the inked surface for pure DCIS.

### Excision Volume

The volume of tissue excised as SOC (main specimen and shaves) and guided shaves was determined from pathology reported tissue dimensions. The amount of additional tissue removed after SOC using guided shaves was expressed as the median volume of guided shaves for each patient out of the median volume of total tissue excised during surgery for each patient.

### Diagnostic Accuracy

The diagnostic accuracy of pFGS was determined by comparing each pFGS image (positive or negative) to the histopathological margin assessment (tumor vs no tumor) at that site. Because histopathology of the imaged tissue was not always available (eg, a guided shave was not taken), a hierarchy of truth standards were used for comparison with pFGS as applicable (eFigure 1 in Supplement 3).

Each pFGS image result was compared with this truth standard hierarchy in an orientation-specific basis to classify each image as true positive, false positive, true negative, or false negative. As a comparison to the diagnostic accuracy of pFGS, the ability of standard pathologic margin assessment to predict residual disease in the cavity was determined by comparing the pathologic assessment of margin status (positive or negative) for each orientation on the main specimen with the pathologic assessment (tumor vs no tumor) of the subsequent corresponding shave (SOC, guided shave, or reexcision). Any SOC margin without a subsequent corresponding shave was not included in this analysis because it lacked a truth standard.

### Statistical Analysis

Analysis for continuous variables included mean, median, SD, minimum, maximum, and sample size for the overall sample and for subgroups. Binary variables were described with frequencies and percentages for the overall sample and subgroups. Demographic and baseline characteristics were summarized by overall descriptive statistics for analysis populations. All analyses were performed under good clinical practice standards using SAS version 9.4 (SAS Institute).

## Results

### Patient Demographic Characteristics

**Figure 2.**
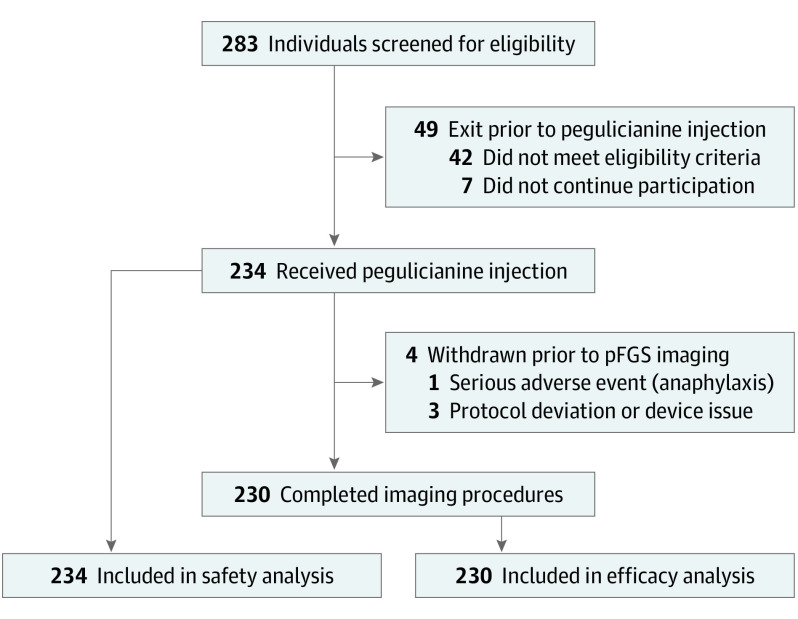
CONSORT Diagram pFGS indicates pegulicianine fluorescence–guided system.

Between February 6, 2018, and April 10, 2020, 234 patients (median [IQR] age, 62.0 [55.0-69.0] years) enrolled in the study according to protocol recruitment goals, and received pegulicianine (Figure 2). Four patients were withdrawn from the study before completing the pFGS procedure but were included in the safety analysis. The remaining 230 patients that completed pFGS procedures were analyzed for performance metrics of the technique. Demographic and tumor characteristics for this cohort are presented in Table 1.

**Table 1.** Demographic and Tumor Characteristics for 230 Patients Who Underwent Pegulicianine Fluorescence–Guided System (pFGS)

Characteristic	No. (%)
Age, median (IQR), y	62.0 (55.0-69.0)
Race^a^	
Asian	15 (6.5)
Black	21 (9.1)
White	183 (79.6)
Multiple races^b^	2 (0.9)
Unknown	9 (3.9)
Ethnicity	
Hispanic	4 (1.7)
Non-Hispanic	216 (93.9)
Unknown	10 (4.3)
Menopausal status	
Post	183 (79.6)
Pre/peri	47 (20.4)
Mammographic breast density^c^	
Almost entirely fatty	12 (5.4)
Scattered areas of fibroglandular density	117 (52.2)
Heterogeneously dense	84 (37.5)
Extremely dense	11 (4.9)
Tumor histology	
Pure DCIS	43 (18.7)
IDC ± DCIS	160 (69.6)
ILC ± DCIS	25 (10.9)
IDC + ILC	2 (0.9)
Largest invasive tumor dimension, mean (SD), cm	1.8 (1.5)
Lymph node status	
At least 1 lymph node (+)	28 (12.2)
All lymph nodes (−)	156 (67.8)
No lymph node biopsy	46 (20.0)
Tumor markers	
Estrogen receptor (+)	209 (91.7)
Progesterone receptor (+)	176 (79.6)
*HER2* (+)	18 (9.8)
Triple negative	10 (4.3)

Abbreviations: DCIS, ductal carcinoma in situ; IDC, invasive ductal carcinoma; ILC, infiltrating lobular carcinoma.

^a^ Race and ethnicity data were included in this study to provide context regarding the study’s generalizability and to comply with mandatory race and ethnicity reporting for studies funded by the NCI. Data were collected via self-report, as recorded in the electronic medical record. Participants were offered multiple choice, as categorized by the electronic medical system, and open-ended answer options. Unknown indicates that patients declined to answer.

^b^ Multiple race includes patient self-report of more than 1 race.

^c^ Defined according to the American College of Radiology Breast Imaging Reporting & Data System.

### Safety Outcomes

Safety assessments were conducted for all 234 patients who received pegulicianine. One participant experienced a serious adverse event of anaphylaxis during the administration of pegulicianine, was treated, recovered without sequelae, and continued with surgery as planned. This individual had a history of allergy to iodinated contrast agents, a condition that did not meet the exclusion criteria at the time of enrollment. After this event, the eligibility criteria were revised to clarify the exclusion of participants with history of allergic reactions to contrast agents or polyethylene glycol (a component of pegulicianine). Following this protocol change, no other serious adverse events were observed in this study. Three other adverse events were deemed likely associated with the study intervention: superficial thrombophlebitis, transient transaminitis, and posttraumatic stress disorder (observed in the patient who experienced anaphylaxis).

### Association of pFGS Imaging and Shave Margin Pathology

Overall, 230 patients completed SOC BCS followed by pFGS imaging and 1584 individual margins with corresponding shave margins were available for analysis (Table 2; eFigure 2 in Supplement 3). Detailed comparison of pFGS lumpectomy cavity imaging to corresponding oriented margins was performed on a per-margin basis using the truth standard hierarchy approach (eFigure 1 in Supplement 3). There were 1091 negative images, 1072 of which were true negatives (98% negative predictive value; Table 3). Of 62 total positive margins, pFGS imaging was positive in 43 (69.4% sensitivity). On a per-margin basis, the overall false-negative rate of the pFGS technology, defined as the proportion of false-negative images (19) of total images (1522), was 1.2% and the per-margin sensitivity was 69.4% compared with 38.2% for routine pathology assessment of the main lumpectomy specimen. This excellent false-negative rate must be considered in the context of a relatively low per-margin specificity of 70.4%, which reflects the low positive predictive value of the technology.

**Table 2.** Patients With Positive Margins After Standard of Care (SOC) Stratified by Surgical Technique

SOC approach	No (%) [95% CI]			
	Total (N = 230)	SOC		No SOC shaves (n = 86)
		Comprehensive shaves (n = 58)^a^	Selective shaves (n = 86)^b^	
Patients with positive margins after SOC	38/230 (16.5) [12.0-22.0]	11/58 (19.0) [9.9-31.4]	15/86 (17.4) [10.1-27.1]	12/86 (14.0) [7.4-23.1]
pFGS identified at least 1 positive SOC margin	16/38 (42.1) [26.3-59.2]	6/11 (54.5) [23.4-83.3]	4/15 (26.7) [7.8-55.1]	6/12 (50.0) [21.1-78.9]
pFGS identified all positive SOC margins	11/38 (28.9) [15.4-45.9]	4/11 (36.4) [10.9-69.2]	3/15 (20.0) [4.3-48.1]	4/12 (33.3) [9.9-65.1]
pFGS-guided conversion of positive SOC to final negative margins	6/38 (15.8) [6.0-31.3]	2/11 (18.2) [2.3-51.8]	1/15 (6.7) [0.2-31.9]	3/12 (25.0) [5.5-57.2]
Patients with final positive margins	35/230 (15.2) [10.8-20.5]	10/58 (17.2) [8.6-29.4]	16/86 (18.6) [11-28.4]	9/86 (10.5) [4.9-18.9]

Abbreviation: pFGS, pegulicianine fluorescence–guided system.

^a^ Comprehensive SOC shaves refer to additional tissue being removed from all surfaces of the cavity walls after resection of the main specimen but before pFGS imaging.

^b^ Selective SOC shaves refers to additional tissue being removed from specific locations in the cavity walls after resection of the main specimen but before pFGS imaging based on intraoperative imaging, palpation, pathology, etc.

**Table 3.** Diagnostic Accuracy of Pegulicianine Fluorescence–Guided System (pFGS) for Predicting Residual Cancer in the Lumpectomy Cavity: Margin Level Analysis (N = 230 Patients; 1584 Measurements)

Diagnostic method	No./total No. (%) [95% CI]			
	Sensitivity	Specificity	Predictive value	
			Positive	Negative
pFGS^a^	43/62 (69.4) [56.3-80.4]	1072/1522 (70.4) [68.1-72.7]	43/493 (8.7) [6.4-11.6]	1072/1091 (98.2) [97.3-98.9]
Pathology assessment of main specimen margin^b^	29/76 (38.2) [27.2-50.0]	445/488 (91.2) [88.3-93.5]	29/72 (40.3) [28.9-52.5]	445/492 (90.5) [87.5-92.9]

^a^ Each pFGS image result was compared with a truth standard hierarchy.

^b^ Each margin orientation on the main lumpectomy specimen was compared with the pathologic assessment of the subsequent corresponding shave.

### Association of pFGS Imaging and Final Margin Status

The study protocol required up to 2 additional shaves in a single margin orientation for a positive pFGS signal. In the overall study cohort, 37 patients with positive pFGS did not have an additional excision performed for a positive pFGS signal; however, not all of these cases were assessed to be protocol violations. According to the study protocol, no protocol violation occurred if there was insufficient tissue available for excision (eg, too close to skin or nipple or at the chest wall) or if the protocol shave limit of 2 per orientation had been reached. After detailed review of the data, only 12 patients were deemed to have had protocol violations because the shave had not been removed when indicated by pFGS. These instances were assessed according to the hierarchical approach for imaging-pathology association (eFigure 1 in Supplement 3).

Negative margins after SOC BCS were achieved in 192 of 230 patients (83.5%). Within this group, use of pFGS resulted in additional pFGS-guided excisions in 115 patients, 14 of whom were found to have residual tumor in the additionally excised margins. Eleven had negative margins at final pathology.

Positive margins after SOC BCS were identified in 38 of 230 patients (16.5%). Among these patients, 29 had positive pFGS in at least 1 orientation. Of these patients, 23 had additional pFGS-guided excisions, and 12 of these patients had residual tumor found in the final pFGS-directed margins. Overall, 138 of 230 patients (60%) had additional excisions guided by pFGS and residual tumor was found in 26 of 138 (19%) of those patients. On a per-patient level, the false-negative rate of pFGS was 23.7% (9 of 38) with a patient-level sensitivity of 76.3% (29 of 38) (eTable 3 in Supplement 3).

### Association of pFGS With Reexcision Rate

In the overall cohort, 28 of 230 patients (12.2%) proceeded with a second procedure for positive margins, consisting of 24 reexcisions and 4 mastectomies. Reexcision would have been indicated after SOC surgery in 38 patients (16.5%) for positive margins. Nine patients had a positive final margin but negative pFGS signal. Of these, 7 had a second surgery and tumor was found in 3 of those patients. The remainder of patients with positive margins after SOC surgery had positive pFGS imaging; 23 had additional pFGS-guided shaves based on imaging, and a final negative margin was achieved in 6 patients. An additional 6 had a positive signal but pFGS-guided excisions were not taken based on surgeon judgment (eFigure 3 in Supplement 3).

Among the 32 patients who were treated per protocol with intraoperative reexcision for positive imaging, pFGS reduced the need for reexcision in 6 of 32 patients, averting the need for second surgery by 19%. Among all patients with positive pFGS signal, adherence to per-protocol intraoperative reexcision could potentially have prevented reexcisions in at least 10 and up to 12 of 38 patients (26.3% to 31.6%) with positive margins after SOC surgery.

### Association of pFGS With Identification of Other Breast Pathology and Excision Volume

Of the 230 participants who completed the pFGS procedures, a total of 243 guided shaves were excised at a mean (SD) rate of 1.1 (1.2) guided shaves per patient; 34 pFGS shaves contained cancer, 100 had benign mammary tissue only, and 209 contained benign histologies (eTable 2 in Supplement 3). The median per patient volume of pFGS shaves was 4.0 (95% CI, 0.0-102.8) cm^3^ compared with 57.8 (95% CI, 11.5-252.7) cm^3^ for lumpectomy volume (main specimen) and 65.4 (95% CI, 17.9-276.9) cm^3^ for total SOC tissue volume (main specimen plus SOC shaves). The median contribution of the pFGS shaves to the total resection volume was 6.5% (0.0-55.9) (eTable 4 in Supplement 3).

## Discussion

In this nonrandomized controlled trial, among patients undergoing BCS, positive surgical margins were associated with local recurrence at the tumor bed.^22,23^ Current SOC BCS, largely guided by palpation and specimen radiography, does not consistently identify residual disease in the lumpectomy cavity, potentially leading to incomplete tumor removal and additional surgical procedures. A preferred approach would be to evaluate the entire lumpectomy cavity intraoperatively following SOC excision to allow real-time guided excision of residual tumor in the cavity walls, with intraoperative verification of no residual tumor. This would reduce treatment delay, pain, anxiety, and health care costs associated with second surgeries. This approach could also provide an opportunity to improve cosmetic outcomes by reducing reexcision rates and allowing the surgeon to take smaller lumpectomy specimens without compromising oncologic outcomes.

The current study built on the findings of a previously reported single-center, proof-of-concept trial of pFGS.^20,21^ In the current multicenter feasibility study, the tumor detection algorithm was tested, hands-on user training of surgical staff was completed, and site-specific workflow needs were identified and addressed to expand the potential for technology implementation across multiple sites. This study provides evidence that the use of pFGS following SOC lumpectomy procedures can identify residual cancer missed during the initial BCS, with 19% of pFGS-guided excisions containing residual tumor. Additionally, 14 of 192 patients (7%) who had negative margins following SOC excision were found to have residual cancer in the pFGS-guided shaves and would have not received additional surgery to remove the residual cancer in the absence of pFGS. Although this did not impact reexcision rate, these patients may have benefited from a more complete excision by a more complete eradication of residual disease.

pFGS identified positive signal in the cavity of more than half of patients with positive margins after SOC, although not all pFGS positive signals corresponded with positive margins. Some of this discrepancy may be because of limitations in SOC margin pathology accuracy to predict residual cancer in the cavity.^24^ We discovered that pFGS achieved a high negative predictive value but a lower positive predictive value. These performance characteristics allow for a lower rate of missed residual disease. Moreover, these findings suggest a potential future role for pFGS in instances where surgeons may reconsider the need for reexcision surgery in patients with a close margin where the pFGS scan was negative in that cavity orientation.

An earlier study using pFGS showed that false-positive readings occurred more frequently in orientations with close prior margins.^25^ Comparing sensitivity of pFGS and main specimen margin assessment provides useful context for the diagnostic accuracy of the different approaches. On a per margin analysis, the sensitivity of pFGS (69.4%) was a notable improvement over SOC assessment alone (38.2%) for predicting the presence of residual tumor in the cavity. This result is consistent with the poor sensitivity previously reported for lumpectomy margin status to predict residual cancer.^24^ However, this comparison alone does not consider the important fact that pFGS provided residual tumor assessment in real time during the surgery, while the routine main specimen margin assessment did not. This sensitivity comparison is further limited because pathologic assessment of the imaged tissue or subsequent cavity margin was not always available for direct comparison (eg, a guided shave was not taken) and thus a hierarchy of truth standards was used to evaluate performance of this technology (eFigure 1 in Supplement 3).

In context, the low positive predictive value of the pFGS approach resulted in 6.1% (4.0 cm^3^) more tissue removed from 1.1 guided shaves per patient. This additional tissue would likely have minimal impact for cosmesis and would compare favorably with reexcision surgery for positive margins. We anticipate that pFGS has the potential to minimize the amount of benign tissue removed relative to SOC, particularly when compared with the comprehensive shave technique.^14^

Overall, pFGS reduced the need for second surgeries in 6 of the 38 patients with positive margins after SOC BCS. However, an additional 6 patients may have potentially avoided reexcision if an additional pFGS shave had been performed intraoperatively as would have been indicated by the intraoperative imaging. Among patients who underwent additional intraoperative excisions of Lumicell-positive margins per protocol, the need for second surgery was reduced by 19%, which could be a clinically meaningful improvement in reexcision rate.

As described above, after the anaphylactic reaction and subsequent protocol eligibility criteria modification to exclude participants with preexisting allergies to contrast agents, no other anaphylactic reactions were observed in this study. This was the only serious adverse event determined to be related to the study drug in more than 342 patients who received a pegulicianine injection in our published and unpublished feasibility studies.^19,20,21^ The pegulicianine safety profile compares favorably with isosulfan blue, routinely used in BCS for sentinel lymph node mapping, which has an allergic reaction rate of 1% to 3%.^26,27,28,29^

### Limitations

During this feasibility and training study, we encountered protocol deviations that may have impacted the overall performance of pFGS. Of 230 participants, 175 (68%) had positive pFGS images following completion of SOC, but in 37 of these patients no pFGS shaves were removed either because of protocol deviation or because the protocol limited the number of additional shaves that could be excised at any single margin. While we were still able to demonstrate utility of pFGS in the BCS setting in detection of residual disease, these protocol deviations may have precluded full pathologic correlation and evaluation of clinical utility for many of the pFGS images and may have prevented conversion of positive margins to negative margins in some patients. These challenges informed strategies to improve standardization and selection of end points for a definitive prospective randomized pivotal study in which the clinical value of pFGS will be more definitively evaluated, both for the detection of residual disease and for potential reduction of reexcision rates. Moreover, the observations and learnings from this study were incorporated into the protocol and training program for this study.

## Conclusions

This multicenter feasibility study of pFGS in 234 patients undergoing BCS found that pegulicianine had an excellent safety profile and reduced the reexcision rate in those who underwent additional pFGS-guided excisions. With its high negative predictive value, pFGS is a promising intraoperative tool that has potential to detect residual disease and possibly reduce the rate of reexcision, thereby improving surgical and cosmetic outcomes in patients undergoing BCS for early-stage breast cancer.

## Notes

Supplement 1. Trial protocol

Supplement 2. Statistical analysis plan

Supplement 3. eFigure 1. Definition of truth standards used to evaluate pFGS image result or SOC margin assessment at identifying tumor in the corresponding cavity orientation eFigure 2. pFGS Outcomes Flowchart eFigure 3. Final margin status of patients with positive margins following SOC excision, candidates for re-excision procedure (n=38) eTable 1. Participating site location and enrollment eTable 2. Tissue Type Distribution in pFGS-guided shaves eTable 3. Diagnostic accuracy of the pFGS system for predicting residual cancer in the lumpectomy cavity: patient level analysis (n=230 patients) eTable 4. Excisional volumes stratified by surgical technique

Supplement 4. The INSITE study team

Supplement 5. Data sharing statement
