# Supplementary material for: Clinical Impact of Intraoperative Margin Assessment in Breast-Conserving Surgery With a Novel Pegulicianine Fluorescence–Guided System: A Nonrandomized Controlled Trial
Source: JAMA Surg. 2022 May 11;157(7):573–80. doi: 10.1001/jamasurg.2022.1075 (PMC9096689; doi:10.1001/jamasurg.2022.1075)
Supplement: Supplement 1. — Trial protocol [file jamasurg-e221075-s001.pdf]

Study number: LUM-015/2.6-001

Protocol number: CL0006

Initial Protocol Version A: 21 June 2017
Protocol Version B: 14 June 2018
Protocol Version 03: 07 December 2018
Protocol Version 04: 15 February 2019

Title: FEASIBILITY STUDY PHASE C: EXPANSION INTO MULTIPLE INSTITUTIONS FOR TRAINING IN THE USE OF THE LUM IMAGING SYSTEM FOR INTRAOPERATIVE DETECTION OF RESIDUAL CANCER IN THE TUMOR BED OF FEMALE SUBJECTS WITH BREAST CANCER.
Grant # 1R44CA211013-01

System: LUM Imaging System: LUM015 imaging agent and LUM Imaging Device
IDE # G140195

Device and Imaging agent supplier
Lumicell, Inc.
Contact information:
Felix Geissler, M.D., Ph.D.
Chief Medical Officer
felix@lumicell.com
Phone: 609-498-4809

Protocol Lead Principal Investigator:
Barbara L. Smith, MD, PhD
Director, Breast Program
Massachusetts General Hospital
55 Fruit Street
YAW9
Boston, MA 02114
Phone: 617-726-6500
Fax: 617-724-1079

Medical Monitor: Karl Gallegos, MD
DSMB Contact: Robert Swain, PhD

LUMICELL CONFIDENTIAL

This document is confidential. Do not disclose or use except as authorized.

LUMICELL CONFIDENTIAL
This document is confidential. Do not disclose or use except as authorized.

TABLE OF CONTENTS

1 PURPOSE....4

2 SUMMARY OF OBJECTIVES....4

3 BACKGROUND....4

3.1 Study disease....4

3.2 Rationale....4

4 PRODUCT DESCRIPTION....5

4.1 Intended use....5

4.2 Indications for use....6

4.3 LUM015 imaging agent....6

4.4 LUM Imaging Device....7

4.5 LUM decision software....7

5 STUDY DESIGN....8

5.1 Overview and background....8

5.2 Definitions....9

5.3 Main lumpectomy specimen and shaved cavity margins nomenclature....10

5.4 Phase C study description....11

5.5 Schema....15

6 SUBJECT SELECTION....15

6.1 Eligibility criteria....15

6.2 Exclusion criteria....16

6.3 Inclusion of women, minorities and other underrepresented populations....18

7 SUBJECT ENROLLMENT....18

7.1 General guidelines for screening and enrollment....18

7.2 Enrollment process....18

8 INTERVENTIONAL PLAN....18

8.1 LUM015 administration....18

8.2 Imaging of the lumpectomy cavity walls and shaved cavity margins....19

8.3 General concomitant medication....20

8.4 Duration of therapy....20

8.5 Duration of follow-up....20

8.6 Criteria for removal from study....20

8.7 Criteria for stopping the study....21

This document is confidential. Do not disclose or use except as authorized.

9 LUM015 FORMULATION AND ADMINISTRATION 22
9.1 Description 22
9.2 Form 22
9.3 Storage and stability 22
9.4 Compatibility 22
9.5 Handling 22
9.6 Availability 22
9.7 Preparation 22
9.8 Administration 23
9.9 Ordering 23
9.10 Accountability 23
9.11 Receipt and return of LUM015 and the LUM Imaging Devices 23
10 STUDY CALENDAR 25
11 SAFETY 25
11.1 LUM015 pharmacokinetics 26
11.2 Expected adverse events (associated with LUM015) 26
11.3 Expected adverse device effects (associated with the LUM Imaging System) 28
12 ADVERSE EVENT REPORTING REQUIREMENTS 28
12.1 Definitions 28
12.2 Procedures for recording and reporting safety 30
12.3 Reporting requirements 30
12.4 Reporting to Lumicell 30
12.5 Reporting to the Institutional Review Board (IRB) 31
12.6 Reporting to the Food and Drug Administration (FDA) 31
12.7 Reporting to hospital risk management 32
12.8 Monitoring of adverse events/adverse device effects and period of observation 32
12.9 Protocol deviations 32
13 DATA AND SAFETY MONITORING 33
13.1 Data reporting 33
13.2 Safety review 33
13.3 Monitoring 33
13.4 Data Safety Monitoring Board 34
14 REGULATORY CONSIDERATIONS 34
14.1 Protocol review and amendments 34
14.2 Informed consent 34

This document is confidential. Do not disclose or use except as authorized.

14.3 Ethics and Good Clinical Practice (GCP) 34
14.4 Study documentation 35
14.5 Records retention 36

15 STATISTICAL CONSIDERATIONS 36
15.1 General methods 36
15.2 Sample size 37
15.3 Populations for analysis 37
15.4 Exclusions 37
15.5 Efficacy and intraoperative imaging data analysis 37
15.6 Safety analysis 38

16 PUBLICATION PLAN 38

17 REFERENCES 38

18 APPENDICES 40

This document is confidential. Do not disclose or use except as authorized.

1 PURPOSE

We recently completed Phase A and Phase B of our Feasibility Study of the LUM Imaging System (NCI funding 5R21CA173762-02): Feasibility clinical study of real-time tumor bed imaging in breast cancer patients. Based on the experience gained from this work, we propose to expand the Feasibility Study into multiple institutions (Phase C) with the main goal of standardizing the training for surgeons and clinical staff and to collect additional imaging and pathology data to further refine and verify the tumor detection algorithm. Phase C of the Feasibility Study will be expansion to a maximum of 20 sites, with each site having approximately 1-3 surgeons participating. The study may include up to 250 patients. This study remains non-randomized and open label.

2 SUMMARY OF OBJECTIVES

- Collect data to refine and verify the tumor detection algorithm
- Complete hands-on training of the surgeons and clinical staff that will be participating in the pivotal study to evaluate the efficacy and safety of the LUM Imaging System in breast cancer surgeries
- Identify and address any site-specific or user-specific issues for using the LUM Imaging System in breast cancer surgeries
- Collect safety and efficacy data

3 BACKGROUND

3.1 Study disease

For breast cancer lumpectomies, the presence of residual cancer cells left in the tumor bed after initial resection is inferred by post-operative margin assessment of the resected tissue by a pathologist. For invasive carcinoma, a positive margin is defined as having tumor present at the inked side of the outermost surface of the lumpectomy specimen [1]. When ductal carcinoma in situ (DCIS) is present, a positive margin occurs when DCIS is found within 2 mm from the inked surface [2]. Positive lumpectomy margins are the most important risk factor for local recurrence of breast cancer [1-5] and dictate that a second surgical procedure be performed to obtain tumor-free margins. The rates of close or positive margins have been reported between 17 to 59% [6-10]. Among the more than 318,000 women expected to be diagnosed with breast cancer in the US in 2017, approximately 200,000 will undergo lumpectomy and approximately a third will require additional surgery for positive margins. Thus, a diagnosis of positive margins indicates that additional treatments are needed. This places a heavy burden on patients and adds significant cost to the healthcare system.

3.2 Rationale

Achieving negative margins during tumor excision is critical to assure that all or most of the cancer has been removed from the patient. Current standard of care dictates that tissue removed during surgery is analyzed post-operatively for tumor histology and margin assessment by a pathologist as described above. This procedure is time consuming, often requiring 7-10 days of pathology

LUMICELL CONFIDENTIAL

This document is confidential. Do not disclose or use except as authorized.

testing after the operation before margin status is known. In addition, this process is prone to sampling errors, as only a finite number of tissue slices can be examined, meaning that only a small fraction of the lumpectomy margin surface is assessed, < 1% of lumpectomy surface area by some estimates. Thus, a safe method to directly assess the entire tumor bed intraoperatively and identify residual microscopic disease for immediate resection intraoperatively would be highly beneficial for patients.

Lumicell has developed the LUM Imaging System, which consists of a fluorescence-based imaging agent (LUM015), a hand-held, wide-field detector (LUM Imaging Device) that can image the lumpectomy cavity in seconds, and a tumor detection algorithm that highlights regions suspected to contain cancer and displays them on a computer monitor. The LUM Imaging system is intended to be used to scan the lumpectomy cavity walls intraoperatively after the resection of the main lumpectomy specimen.

The Lumicell Imaging System may enable surgeons to achieve negative margins during the initial surgery and eliminate or reduce the need for re-excision surgeries when compared with current standard of care procedures. Lumicell's system images the entire lumpectomy cavity surface in vivo, overcoming tissue handling and sampling limitations of standard pathology analysis of resected tissue. The LUM Imaging System provides real-time feedback to the surgeon about the status of the lumpectomy cavity walls, and does not require patient immobilization; hence, the system may not significantly impact surgical workflow.

The LUM Imaging System has been tested in a two-phases of the Feasibility Study at the Massachusetts General Hospital. Fifteen patients in Phase A and 45 patients in Phase B were included in the study. The results indicate zero false negatives in the margin cavity walls and avoidance of two second surgeries based on the detection of positive signal with the LUM Imaging Device. These results are presented in the Phase B report.

This experience showed that user related issues are not easily identified until the surgeon uses the system in the clinical setting. No amount of "dry runs" outside the operating room reveal the important issues that arise during surgery; for example, optimizing a surgeon's approach for keeping the hand-held unit in contact with the tissue, thorough scanning of the cavity walls, and co-location of the image with the additional shaved margin tissue to be removed. Therefore, we believe that it is critical to introduce the LUM Imaging System into additional clinical sites and train the clinical staff prior to the pivotal study.

4 PRODUCT DESCRIPTION

4.1 Intended use

The LUM Imaging System is a combination product consisting of the LUM015 imaging agent and the LUM Imaging Device. LUM015 is administered to the patient via intravenous injection 4 ± 2 hours prior to surgery. The LUM Imaging Device is intended for use in vivo following the excision of the main lumpectomy specimen to potentially help determine whether cancer remains in the lumpectomy cavity walls based on the tissue fluorescence signals.

This document is confidential. Do not disclose or use except as authorized.

4.2 Indications for use

The LUM Imaging System is indicated for use in patients undergoing a breast conserving surgery (lumpectomy) to remove breast cancer. The LUM Imaging System is intended to be used whenever breast tissue is removed, histopathology evaluation of the tissue is the standard of care and/or it is essential that the tissue margins be examined for completeness of removal using standard surgical procedures. The LUM Imaging System is used in vivo following the excision of the main lumpectomy specimen to assist in locating residual abnormal tissue, therefore, facilitating tumor resection and reducing the risk for cancer-positive margins in the resected tissue.

4.3 LUM015 imaging agent

Lumicell engineered LUM015, a fluorescence-based imaging agent that accumulates within and around cells. In regions enriched with cathepsin enzymes, such as cancer cells, LUM015 is altered to produce a fluorescent signal. In its nominal state, the fluorescence of LUM015 is suppressed by an internal quencher molecule (QSY21). The presence of cathepsin enzymes is not fully confined to cancer cells; hence, data from this study will help determine the cancer localization properties of LUM015. Once cathepsin enzymes cleave LUM015 at its amino acid backbone, the quencher is released and the fluorescent dye (Cy5) in LUM015 emits detectable fluorescence (**Figure 1**). The absorption and emission wavelength maxima for Cy5 are 649 nm and 670 nm respectively. LUM015 employs a fluorescent molecule with excitation and emission in the far red spectrum (< 700 nm wavelength) because in that range light can travel effectively through 1mm of tissue and tissue autofluorescence is minimal, allowing higher sensitivity due to lower background [11].

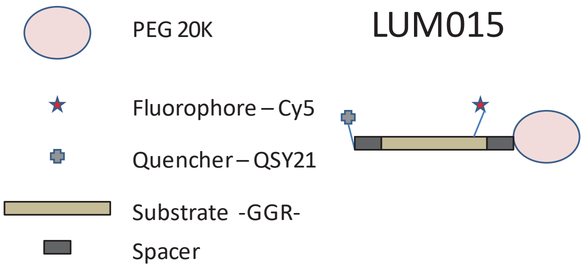

Figure 1: Schematic representation of LUM015.

Cathepsins are a family of enzymes that are involved in the degradation of the extracellular matrix to allow tumor growth and progression. These enzymes are upregulated in most human cancers and are also present in tumor associated macrophages at the tumor's invasive front [12-16]. Platt et. al. showed 60-fold higher cathepsin activity in breast cancer tissue than in healthy breast tissue [17]. Others report over-expression of cathepsin enzymes (B, K, L and D) in ductal carcinoma in situ as well as in invasive carcinomas (lobular and ductal) [13, 17-20]. Also, cathepsin B is typically over-expressed in inflammatory breast cancer, one of the most lethal forms of primary breast cancer. [21, 22]. The peptide sequence used in LUM015 is a pan-cathepsin substrate meaning that it will be cleaved by multiple enzymes of the cathepsin family. Because high activity of cathepsin enzymes is found in tumor cells and tumor associated macrophages surrounding the tumor at the invasive front, these enzymes provide an excellent marker for activating LUM015 at the tumor margin. The fluorescence of LUM015 is then detected with the LUM Imaging System

LUMICELL CONFIDENTIAL

This document is confidential. Do not disclose or use except as authorized.

to identify cancer and cancer-related cells for immediate resection and to distinguish them from adjacent normal tissue.

4.4 LUM Imaging Device

The LUM Imaging Device consists of a computer control unit, monitor and light source mounted on a cart, and a hand-held optical head (LUM002/LUM003 imaging head). The computer control unit collects, analyzes (based upon Lumicell's detection algorithm) and displays the resulting images gathered by the imaging head in real-time. The light source provides the illumination to excite the fluorescent dye present in LUM015. Light is transferred from the light source to the imaging head using an optical fiber bundle. The imaging head (**Figure 2**) was designed as a lightweight hand-held tool with a small profile to allow easy maneuverability and limited intrusiveness in the operating room. Because incisions and lumpectomy cavities can vary in size, Lumicell offers two options for the distal end diameter: LUM002, which has a 2.6 cm field of view along with a 3.1 cm outer diameter and LUM003, which has a 1.3 cm field of view and a 1.9 cm outer diameter. The surgeons will be able to choose which device size to use for any given surgery. These two device options were proven to be optically equivalent when imaging freshly excised breast tissue. Both imaging heads have a 45° bend at the distal end for examination of the walls of the lumpectomy cavity.

The hand-held optical head will be used within the sterile surgical field. Consequently, a sterile barrier assembly will be provided to cover the optical head, which comes into contact with both the surgeon and the patient's exposed tumor bed. The sterile barrier assembly is installed on the LUM Optical Head in the OR using aseptic procedures.

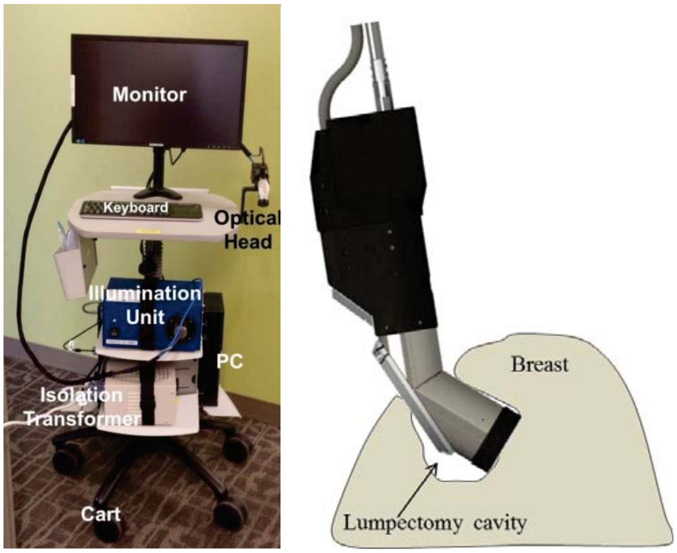

Figure 2: (Left) Photo of the LUM 2.6 Imaging System. (Right) Rendering of the LUM002 Optical Head scanning a lumpectomy cavity. The sterile cover over the device is not shown.

4.5 LUM decision software

The LUM Imaging System is powered by Lumicell's proprietary decision software. The decision software uses an initial set of images acquired from the lumpectomy cavity after the main specimen is removed to set the tumor detection threshold. Then, while the surgeon scans the lumpectomy cavity walls with the LUM Optical Head, the decision software compares the intensity of an image against the tumor detection threshold and identifies whether a region of the image is suspected to contain tumor. Regions with fluorescence signal above the threshold are highlighted in the computer monitor for the surgeon to see in real-time; that is, there is no delay between the scanning of a region and the display of the results from that region. **Figure 3** shows the user interface and what the surgeon sees during scanning of the cavity walls. The decision software was developed and optimized during Phase A and Phase B of the Feasibility Study in breast cancer patients.

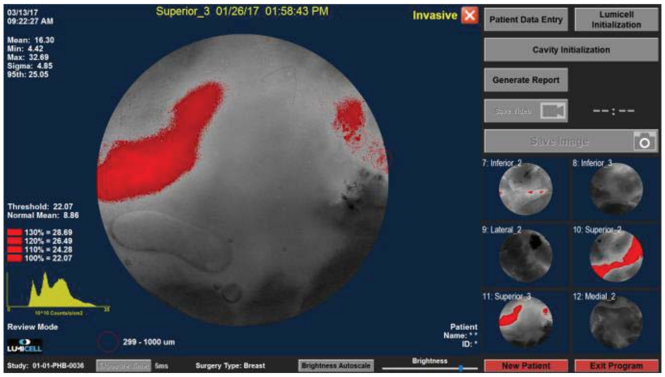

Figure 3: Screen shot of the user interface for the LUM decision software. Regions highlighted in red indicate areas suspected to have tumor.

5 STUDY DESIGN

5.1 Overview and background

Phase A and Phase B of the Feasibility Study were conducted at the Massachusetts General Hospital (MGH). Data from these studies were used to develop the Lumicell tumor detection algorithm that is implemented in the Lumicell decision software. Phase A included 5 subjects not injected with LUM015 for assessment of tissue autofluorescence and 10 subjects receiving LUM015 (5 subjects at a dose of 0.5 mg/kg and 5 subjects at a dose of 1.0 mg/kg) to assess the ability to distinguish LUM015 from autofluorescence. Phase A concluded with the analysis of the data to (1) assess patient safety, (2) determine initial parameters for the tumor detection algorithm and (3) select the dose of 1.0 mg/kg for Phase B. In Phase B, the tumor detection algorithm was fine-tuned for in vivo detection of residual cancer to guide the resection of additional shaved cavity margins (SCMs), known as therapeutic shaves. There were zero false negatives in the margin cavity and two second surgeries were avoided because the surgeon removed additional shaves guided by the LUM Imaging System that converted a positive margin into a negative one. The detailed results are presented in the Phase B Report.

Two of the three surgeons participating in Phase B requested an optical head with a smaller diameter because the incisions they typically make are too small for the optical head with a 3.1 cm outer diameter to fit. For Phase B, these two surgeons opted to make a larger incision than usual to fit the LUM Optical Head. In response to this request, Lumicell designed and built a smaller

This document is confidential. Do not disclose or use except as authorized.

diameter distal end for the LUM Optical Head; however, the smaller version was not used in Phase B because the study was completed prior to obtaining IRB approval for using this version of the LUM Optical Head in the operating room.

We also refined our training process to highlight information that surgeons found important for using the LUM Imaging System comfortably and as intended. Examples of areas addressed during user training include: (1) making sure that the device is in contact with the tissue being scanned, (2) translating the region identified by the decision software as having cancer into the actual location of the shave to be removed once the LUM Optical Head is out of the cavity, (3) ensuring that the naming of the therapeutic shaves for pathology analysis matches the naming of the Lumicell images, and (4) selecting the proper size of the LUM Optical Head to be used for a given surgery.

The primary objective of this Phase C study is to further refine and verify the algorithm used for detection of residual cancer tissue. The secondary objective is to implement learning from Phases A and B of the Feasibility Study at MGH, in particular, training of breast cancer surgeons and hospital staff at the different sites. We are planning to include up to 20 sites participating in Phase C with approximately 1-3 surgeons at each site. In Phase C, a uniform method for orienting and inking the main specimen will be implemented prior to the subsequent pivotal study (see Appendix B).

5.2 **Definitions**

5.2.1 Definition of positive margins

For this protocol, positive margins are defined using the latest consensus from the Society of Surgical Oncology as follows:

- For invasive cancer with or without associated carcinoma in situ: cancer cells present on ink [1]
- For pure DCIS lesions: DCIS present within 2 mm from the inked surface [2]

5.2.2 Definitions of excised tissue

- **Main specimen**: also known as the “lump” or main mass, refers to the primary piece of tissue removed during a lumpectomy. Typically, this is the largest piece of tissue removed and it is intended to contain most of the tumor.
- **Shaved cavity margins** (SCM): thin pieces of tissue removed from the lumpectomy cavity walls after the main specimen is removed. In this study, we define three different types of SCMs below.
    - **Selective shaves**: shaved cavity margins removed from a specific location of the lumpectomy cavity wall based on intraoperative analysis of the main specimen by methods including, but not limited to, frozen sections, X-ray imaging, ultrasound imaging, palpation or visual examination.
    - **Comprehensive shaves**: shaved cavity margins removed from *all* surfaces of the lumpectomy cavity walls after resection of the main specimen.

LUMICELL CONFIDENTIAL

This document is confidential. Do not disclose or use except as authorized.

- **Therapeutic shaves**: shaved cavity margins removed using the guidance of the LUM Imaging System.

5.2.3 Definition of standard of care breast conserving surgeries

Breast conserving surgeries vary between surgeons and institutions. Table 1 outlines surgical procedure options that will be allowed in this study.

Table 1: Surgical procedures included in Phase C of the Feasibility Study.

SOC procedure	Description	Basis for final margin assessment
A. Main specimen	Main specimen is removed and some selective shaves may be removed based on, but not limited to, palpation, X-ray imaging, visual inspection of the **intact main specimen**. Selective shaves guided by sectioning of the main specimen are not included as part of this procedure (see B below).	Inked surface of main specimen
B. Main specimen followed by selective cavity shaves guided by analysis of main specimen sections	After procedure A above, additional selective shaves are removed based on **analysis of sections from the main specimen**, typically by X-ray imaging	The outermost inked surface for each orientation whether it is from the main specimen or the selective cavity shaves.
C. Main specimen followed by comprehensive cavity shaves	After procedure A above, a cavity shave is removed from all the available orientations. A shave from anterior or posterior may not be removed if no breast tissue remains at these surfaces.	Inked surface of the comprehensive cavity shaves.

5.3 Main lumpectomy specimen and shaved cavity margins nomenclature

Throughout this protocol, reference is made to the orientation of the main specimen, the SCMs and the lumpectomy cavity walls. When a lumpectomy is performed, there are typically 6 surfaces defined for orienting the main specimen. Typically, final margin assessment is made for each orientation. **Figure 4** shows a schematic representation of the nomenclature for the lumpectomy cavity margins for a right-sided breast lumpectomy, showing 5 orientations. The sixth orientation (not shown in the figure) is the “anterior” margin which is the margin nearest to the skin. For surgeons performing selective or comprehensive SCMs, a shave may not be removed from the

This document is confidential. Do not disclose or use except as authorized.

anterior orientation if the lumpectomy cavity extends to the skin or subcutaneous fat. Similarly, a SCM from the posterior orientation (also referred to as "deep") may not be removed if the lumpectomy cavity reaches the fascia over the pectoralis major or serratus anterior muscle. Shaved cavity margins removed under the guidance of the LUM Imaging System will be named "therapeutic shaves" to distinguish them from standard of care SCM. Please see

Appendix **B** for the guidelines on naming the resected tissue.

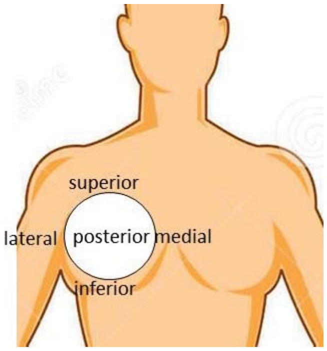

Figure 4: Schematic representation of the surfaces of the lumpectomy cavity walls (white circle). The posterior margin is nearest the fascia of the pectoral muscle. The anterior margin (not shown) is nearest the skin.

5.4 Phase C study description

Data collected during this study will be used to further refine and verify the tumor detection algorithm.

Phase C will consist of up to 5 patients per surgeon, with 1-3 surgeons per site and up to 20 sites. The maximum number of patients participating in the study is 250. The assessment for completing the training will be done in collaboration between Lumicell and each surgeon and will be documented. Surgeons may enroll more than 5 subjects if needed to complete training, or if more subjects are needed to complete the tumor detection algorithm verification.

Subjects undergoing a lumpectomy procedure to treat primary breast cancer will be injected with LUM015 4 ± 2 hours prior to surgery at a dose of 1.0 mg/kg. The sequence of events during the surgical procedure will vary based on the standard of care used by the surgeon (Table 1).

Patients will receive the standard of care practices for each site, including any standard of care shaves. Then, the Lumicell System is used and Lumicell positive tissue should be removed. Please see Appendix B for more details on handling, inking and naming the resected tissue.

In addition, selected institutions will participate in a sub-study as described in Appendix D.

5.4.1 SOC and study procedure

The surgeon will attempt to remove the main specimen with grossly negative margins. The surgeon may remove additional tissue based on intraoperative examination of the main specimen by methods including, but not limited to, palpation (of the main specimen or cavity walls), X-ray imaging, or visual examination. Some surgeons may proceed with comprehensive or selective shaves as part of their standard of care procedure. After any SOC shaves are obtained, the surgeon will conduct the cavity initialization step per protocol and then use the LUM Imaging System to

LUMICELL CONFIDENTIAL

This document is confidential. Do not disclose or use except as authorized.

scan the lumpectomy cavity walls, save images from all the cavity orientations and remove therapeutic cavity shaves as indicated by the Lumicell display. The main specimen, the additional tissue and all SCMs will be handled and named as described in Appendix A and C.

5.4.2 Recording surgeon's decisions

During the LUM imaging procedure, if the surgeon decides to take a different action than the one suggested by the LUM Imaging System, the reason must be documented. The surgeon must notify the computer operator or clinical coordinator at that moment and they will note the reason in the study worksheets. This information will then be transferred to the case report forms. This is of utmost importance for evaluating the impact of the LUM Imaging System in guiding removal of tissue to obtain negative margins. If a shave is not taken when indicated by the Lumicell system, a protocol deviation will be recorded.

5.4.3 Final margin assessment

Final margin assessment for a given orientation is done on the outermost surface resected for that specific orientation (see **Figure 5**). This surface could be on the main specimen, the SOC shaves or the therapeutic shaves. All the resected tissue will be handled and processed for margin assessment following the institution's current practices for breast conserving surgeries.

LUMICELL CONFIDENTIAL

This document is confidential. Do not disclose or use except as authorized.

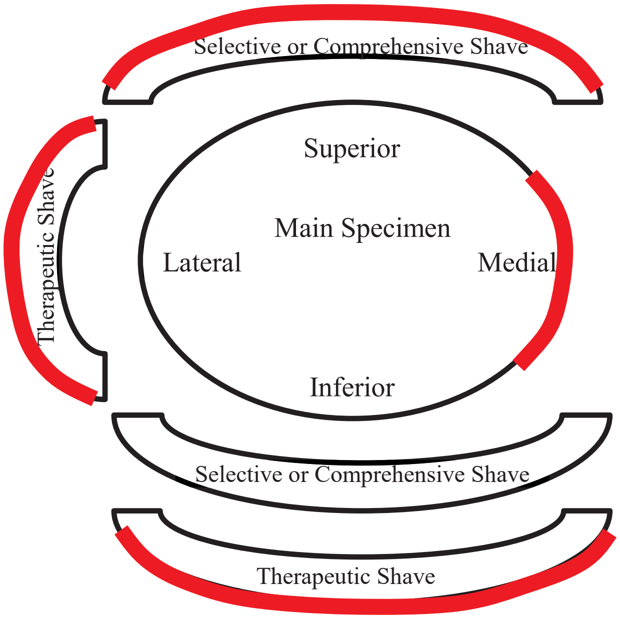

Figure 5: Final margin assessment for a given orientation is based on the outermost surface resected (marked in red in the sketch). For the example in the sketch, final margin assessment for the medial orientation is based on pathology of the main specimen. Final margin assessments for the lateral orientation is based on the therapeutic shave. Final margin assessment for the superior orientation is based on the selective or comprehensive shave. Final margin assessment for the inferior orientation should be based on the therapeutic shave.

5.4.4 Safety observations

All subjects will be observed to assess the safety of LUM015 with standard preoperative, intraoperative and postoperative monitoring from the time of injection to the time they are discharged from the hospital. The subject will have a final safety assessment at the first post-operative visit. All subjects will continue their enrollment and be followed in the study until their medical team determines that no further surgical intervention is required. Further details on follow up are included in section 8.5.

5.4.5 Other general procedures and injection of LUM015

For subjects with non-palpable tumors, an ultrasound, mammogram or MRI-guided localization procedure may be performed prior to or the same day as the surgery. Also, patients having a sentinel lymph node (SLN) mapping procedure may be injected with the radio-tracer Technetium-99 (Tc-99) per standard of care. LUM015 may be injected before or after any of these procedures, as long as it is injected 4 ± 2 hours prior to surgery.

Prior to the surgery, the LUM Imaging Device of the size selected by the surgeon will be positioned in the operating room and an initialization procedure will be conducted to ensure that the performance parameters of the device are within defined specifications. If during the initialization

This document is confidential. Do not disclose or use except as authorized.

procedures, the performance parameters are outside the defined specification, the user is prompted to discontinue use of the device. If the error cannot be fixed within a reasonable time in the operating room, these patients will receive standard of care treatment and will not be imaged with the LUM Imaging Device. These patients will be followed and monitored for safety the same way as those patients that undergo imaging with the LUM Imaging Device. The patients that are not imaged with the LUM Imaging Device will be included in the safety cohort but will not be part of the efficacy cohorts and will not be included in the total number of patients for training purposes.

To avoid excessive tissue resection based on LUM015 signal during this investigational study, surgeons will take no more than 2 therapeutic shaves in a given orientation.

5.4.6 Sentinel lymph node mapping procedures

Dyes used for SLN mapping have fluorescence absorption and emission spectra that overlaps with those from Cy5 (dye conjugated to LUM015). When dyes are used for SLN mapping, they are injected intra-tumoral or peri-tumoral soon after the patient is put under anesthesia and prior to the lumpectomy procedure. Therefore, subjects having injection of dyes for SLN mapping prior to completing the Lumicell imaging procedure are excluded from this study. Recognize that injection of dyes for SLN mapping may occur after completing the Lumicell imaging procedure, but not prior to the imaging procedure.

Patients having SLN mapping procedures may be injected with the radio-tracer Tc-99. In the event that not enough signal is produced from Tc-99, the surgeon may elect to inject a dye to assist in the mapping procedure. If this occurs, these subjects will be excluded from the efficacy cohort but will remain in the safety analysis cohort. The surgeon, at their own discretion, may elect to image the lumpectomy cavity with the LUM System; however, that data will be used as reference only. These subjects will not count towards the total number of patients required to complete the training for that surgeon.

5.4.7 Exploratory study: imaging of sentinel lymph nodes with the LUM Imaging System

In order to explore whether the LUM Imaging System can be used to detect sentinel lymph nodes with or without metastatic cancer, this protocol pursues an exploratory endpoint of collecting images of sentinel lymph nodes. At the surgeon’s discretion, once the sentinel lymph nodes are identified with the radio-tracer, the surgeon can use the LUM Imaging System to image the sentinel lymph nodes in vivo. Regardless of whether the sentinel lymph nodes are imaged in vivo, the surgeon will have the option to image the resected sentinel lymph nodes with the LUM Imaging System in the operating room. Imaging data will be compared against pathology assessment of the resected nodes on whether these contain cancer or not.

LUMICELL CONFIDENTIAL

This document is confidential. Do not disclose or use except as authorized.

5.5 Schema

The schema for Phase C of the Feasibility Study is shown below in Figure 6.

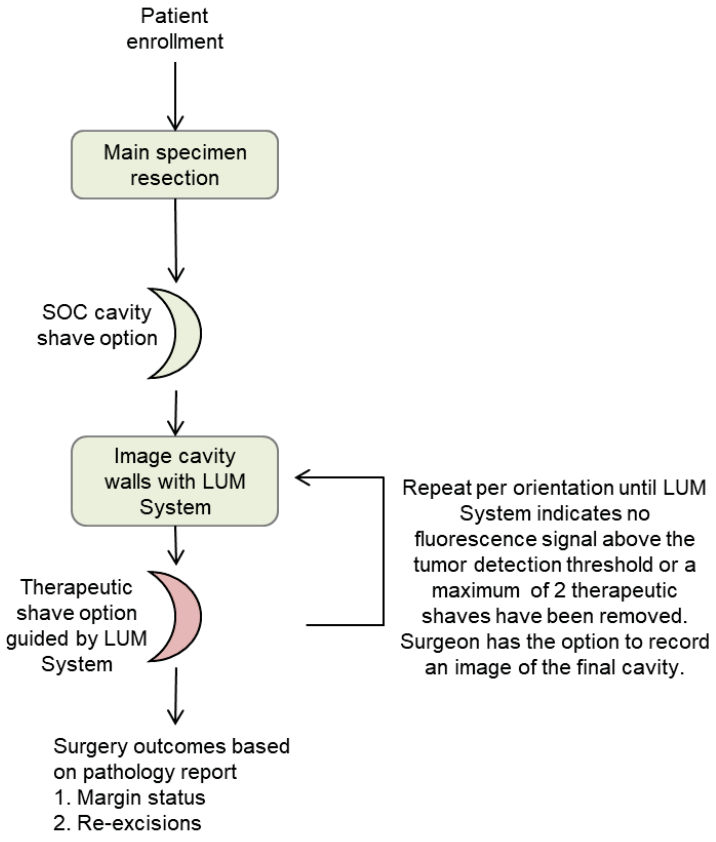

Figure 6: Phase C study schema.

6 SUBJECT SELECTION

6.1 Eligibility criteria

Subjects must meet the following criteria on screening examination to be eligible to participate in the study:

6.1.1 Subjects must have histologically or cytologically confirmed primary invasive breast cancer, ductal carcinoma in situ (DCIS) or a combination of invasive breast cancer and DCIS. The protocol accepted methods for obtaining the histological samples are diagnostic core needle biopsies or fine needle biopsies.

6.1.2 Female, age of 18 years or older. Because no dosing or adverse event data are currently available on the use of LUM015 in subjects <18 years of age, children are excluded from this study.

This document is confidential. Do not disclose or use except as authorized.

6.1.3 Subjects must be scheduled for a lumpectomy for a breast malignancy.

6.1.4 Subjects must be able and willing to follow study procedures and instructions.

6.1.5 Subjects must have received and signed an informed consent form.

6.1.6 Subjects must have no uncontrolled serious medical problems except for the diagnosis of cancer, as per the exclusion criteria listed below.

6.1.7 Subjects must have normal organ and marrow function within limits as defined below:

- Leukocytes ≥ 3,000/mcL
- Platelets ≥ 75,000/mcL
- total bilirubin within normal institutional limits
- AST (SGOT)/ALT (SGPT) ≤ 2.5 X institutional upper limit of normal
- Creatinine ≤ 1.5 mg/dL or creatinine clearance ≥ 60 mL/min/1.73 m^2 for subjects with creatinine levels above institutional normal.

6.1.8 Subjects with ECOG performance status of 0 or 1.

Note: Subjects with a history of multiple drug allergies, atopic subjects, and subjects with atopic syndrome are eligible for the study but should be pre-medicated according to institutional standards prior to injection of the LUM015 imaging agent.

6.2 Exclusion criteria

Subjects who exhibit any of the following conditions at screening will not be eligible for admission into the study.

6.2.1 Subjects who are treated for bilateral breast cancer resection procedure.

6.2.2 Subjects who are pregnant at the time of diagnosis of their breast cancer; this exclusion is necessary because the teratogenic properties of LUM015 are unknown. Because there is an unknown but potential risk of adverse events in nursing infants secondary to treatment of the mother with LUM015, breastfeeding should be discontinued if the mother is treated with LUM015.

6.2.3 Subjects who are sexually active and not willing/able to use medically acceptable forms of contraception (hormonal or barrier method of birth control, abstinence) upon entering the study and for 60 days after injection of LUM015. Should a woman become pregnant or suspect she is pregnant while participating in this study, she should inform her treating physician immediately. Breast cancer patients are routinely advised against becoming pregnant during treatment, so this requirement does not differ from standard of care.

6.2.4 Subjects who have taken an investigational drug within 30 days of enrollment.

This document is confidential. Do not disclose or use except as authorized.

6.2.5 Subjects with prolonged QTc interval defined as greater than 480 ms.

6.2.6 Subjects who will have administration of methylene blue or any dye for sentinel lymph node mapping on the day of the surgery prior to imaging the lumpectomy cavity with the LUM Imaging Device.

6.2.7 Subjects who have not recovered from adverse events due to other pharmaceutical or diagnostic agents.

6.2.8 Subjects with uncontrolled hypertension defined as persistent systolic blood pressure > 180 mm Hg, or diastolic blood pressure > 110 mm Hg; those subjects with known HTN should be stable within these ranges while under pharmaceutical therapy.

6.2.9 History of allergic reaction to polyethylene glycol (PEG).

6.2.10 History of allergic reaction to any oral or intravenous contrast agents.

6.2.11 Uncontrolled intercurrent illness including, but not limited to ongoing or active infection, symptomatic congestive heart failure, unstable angina pectoris, cardiac arrhythmia, COPD or asthma requiring hospitalization within the past 12 months, or psychiatric illness/social situations that would limit compliance with study requirements.

6.2.12 HIV-positive individuals on combination antiretroviral therapy are ineligible because of the potential for pharmacokinetic interactions with LUM015.

6.2.13 Any subject for whom the investigator feels participation is not in the best interest of the subject.

6.2.14 Subjects undergoing a second lumpectomy procedure because of positive margins in a previous surgery prior to entering this study.

6.2.15 Subjects with prior ipsilateral breast cancer surgeries, mastectomies, breast reconstructions or implants.

6.2.16 Subjects who have undergone a surgical biopsy for any reason in the ipsilateral breast performed less than 2 years prior to enrollment of this study.

6.2.17 Subjects with prior ipsilateral reduction mammoplasties (breast reductions) performed less than 2 years prior to enrollment to this study.

6.2.18 Subjects previously treated with systemic therapies to treat the cancer to be removed during this clinical investigation, such as neo-adjuvant chemotherapy or hormonal therapy.

6.2.19 Subjects undergoing breast conserving surgery whose resected specimen will be evaluated with frozen section.

This document is confidential. Do not disclose or use except as authorized.

6.3 Inclusion of women, minorities and other underrepresented populations

As this study is to test the efficacy of an intraoperative imaging technology in female breast cancer subjects, all of the subjects will be women. Males with breast cancer (<1% of breast cancer patients) usually undergo mastectomy procedures and only rarely have lumpectomies, and thus would not be likely to be eligible for this study.

7 SUBJECT ENROLLMENT

7.1 General guidelines for screening and enrollment

An investigator will confirm eligibility criteria and a member of the site study team will complete the protocol-specific eligibility checklist in the Case Report Form.

The site study team will document subject eligibility on the Screening and Enrollment form and notify Lumicell of a potential candidate for the study after the subject has been consented. For circumstances in which a potential candidate does not continue with study treatment or withdraws consent, the site study team should document the subject as “dropped” and document an explanation as to why the subject has not continued with the study.

The subject is not considered enrolled into the study until written Informed Consent is obtained and the investigational product has been administered (Study Day One).

7.2 Enrollment process

The enrollment procedures are as follows:

1. Obtain written informed consent from the subject prior to the performance of any study related procedures or assessments.
2. Complete protocol-specific eligibility checklist and/or the screening questionnaire using the eligibility assessment documented in the subject’s medical/research record. **To be eligible for registration in the study, the subject must meet each inclusion criteria and have none of the exclusion criteria listed on the eligibility checklist.**
3. Assign the subject a study subject ID number. The subject ID numbers at each site should follow the Lumicell assigned format as described at the Site Initiation Visit in compliance with the Case Report Form nomenclature. Numbers should follow a sequential order in accordance with subject enrollment at each site.
4. Notify Lumicell clinical team of eligible subject status and date of proposed surgery.

8 INTERVENTIONAL PLAN

8.1 LUM015 administration

The investigational imaging agent LUM015 will be administered at the hospital 4 ± 2 hours prior to surgery. See section 9 for more details about the preparation and administration of LUM015.

This document is confidential. Do not disclose or use except as authorized.

No investigational or commercial agents or therapies other than those described in this protocol may be administered with the intent to image the subject's malignancy intraoperatively.

As per IV injection standard of care, all subjects will be observed for signs of extravasation or allergic reaction following administration of LUM015 to monitor for adverse pharmacological activity related to the investigational agent. If extravasation is suspected or confirmed, the injection must be stopped immediately and reported to Lumicell. All study safety assessments will continue until the first post-operative visit (approximately 2-3 weeks after surgery).

If there is evidence that there was extravasation of the LUM015 injection, then that patient will be excluded from the efficacy cohort and the cohort used for training the surgeons. However, the patient will be included in the safety data analysis.

If the patient is experiencing an allergic reaction to LUM015 administration, the injection should be stopped immediately, and the following lab values should be obtained:

- Histamine
- Total Blood Complement
- Tryptase levels

The blood draw for each of these tests should be done as soon as possible after the start of the reaction and again at 30 minutes post start of the reaction. If an immediate blood draw is not possible, collect a 30-minute blood draw at minimum. Each blood specimen should be processed, packaged and shipped to the contracted central laboratory for this study. Specific instructions on collection, processing, and shipping can be found in the training materials associated with this study.

In case of an allergic reaction to LUM015, sites should not discard any residual drug remaining in the used syringe. This drug should be stored at -20 degrees C until a Lumicell representative notifies your site of how to proceed with the used syringe.

8.2 **Imaging of the lumpectomy cavity walls and shaved cavity margins**

For Phase C, the surgeon will perform the lumpectomy procedure according to his or her institution's standard of care practice as outlined in sections 5.4.1 to **Error! Reference source not found.**. After the standard of care procedure, the surgeon will have the option of using the LUM002 or the LUM003 depending on their preference and their assessment of likely incision and surgical cavity dimensions. Following the standard of care procedure, the surgeon will image the tumor bed in order to establish a normal tissue baseline for the thresholding algorithm. With the thresholding algorithm applied, the surgeon will then remove therapeutic shaves based on the recommendation of the LUM Imaging System. The surgeon will be limited to removing no more than 2 therapeutic shave for a given orientation (see section 5.3 for definitions of orientations). If the surgeon decides to take a different action than the one suggested by the LUM Imaging System, this is considered a protocol deviation. The surgeon must notify the computer operator or clinical coordinator for documenting the reason for not following the study procedures in the study

LUMICELL CONFIDENTIAL

This document is confidential. Do not disclose or use except as authorized.

worksheets. The surgeon has the option to record an image from the final cavity after the 2 therapeutic shaves are removed.

Also, ex vivo images of the non-margin side of shaved cavity margins will be recorded in the operating room after the in vivo portion of the imaging session is completed.

8.3 General concomitant medication

A complete listing of all medications (including non-prescription, vitamins, herbal products and essential oils) currently being taken on a regular basis prior to surgery will be obtained at screening and updated as needed at enrollment and at follow-up. Concomitant medications will be recorded in the medical record and case report form.

8.4 Duration of therapy

The imaging agent LUM015 will be injected as a single dose 4 ± 2 hours prior to surgery. There is no recurrent administration of the imaging agent. The treatment ends after the surgery is completed. Subjects' participation in the study may be discontinued by the principal investigator at any time for any of the following reasons:

- unacceptable adverse event or adverse device effect
- administrative reasons, such as imaging agent no longer available
- subject noncompliance,
- safety concern,
- subject decides to withdraw from the study, or
- general or specific changes in the subject's condition render the subject unacceptable for further treatment in the opinion of the treating investigator.

8.5 Duration of follow-up

All study interventions will be completed at the end of the surgical procedure. All subjects will continue their enrollment until the first follow-up visit and will continue to be followed in the study until their medical team determines that no further surgical intervention is required. For example, if a patient needs a second surgery because of positive margins, the patient will remain enrolled in the study until the second surgery (whether a mastectomy or a lumpectomy re-excision) is performed, the pathology report is available and her medical team determines that no additional surgery is needed. When attending their first post-operative visit, a blood draw will be collected for a CBC (red blood cells, white blood cells and platelets) and serum chemistry (alkaline phosphatase, total bilirubin, BUN, calcium chloride, glucose, potassium, total protein, SGOT [AST], SGPT [ALT], sodium) tests to assess for possible adverse events. At the time of the visit, the patient will be interviewed to determine any potential adverse events. Subjects with adverse events that are determined to be possibly related to the investigational product will be followed until resolution or stabilization of the adverse event.

8.6 Criteria for removal from study

Subjects will be removed from study when any of the criteria listed in section 6.2 apply. The reason for study removal and the date the subject was removed must be documented in the study-specific case report form (CRF). A subject removed from the study will be given standard of care treatment.

This document is confidential. Do not disclose or use except as authorized.

Subjects removed from the study prior to dosing with LUM015 will not be included in the total patient counts and will be replaced with a new subject.

Individual patients may be discontinued from the study by the Investigator or Lumicell at any time if either determines that it is not in the best interest of the patient to continue (e.g., continuation in the study represents a serious medical risk to the patient). This may include, but is not limited to, the presence of serious, life-threatening adverse events, unanticipated adverse device effects, adverse events, or adverse device effects that are unacceptable in nature, severity, or frequency as assessed by the Investigator.

Patients must be discontinued if they become pregnant or withdraw consent. Patients may be discontinued due to noncompliance with the protocol. While patients will be encouraged to complete the study, they may voluntarily withdraw at any time.

In the event of unusual or life-threatening complications, participating investigators must immediately notify the Medical Monitor, Karl Gallegos, MD, the Principal Investigator, and the Lumicell study monitor.

8.7 **Criteria for stopping the study**

The events listed below are the stopping rules for the study. In the case of any of these events, no additional patients will be recruited, dosed or imaged until the issue is resolved.

- Recommendation by the Data Safety Monitoring Board after evaluation of cumulative safety reviews
- Any death probably or definitely related to the treatment with LUM015 or the Imaging Device.
- Any other event that is deemed unacceptable in nature, severity, or frequency as assessed by the Investigator.
- Allergic reactions of Grade 3 or greater as defined in CTCEA 5.0

In the case that a safety event requiring enrollment suspension occurs, a prompt cumulative review of safety data and the circumstances of the event in question will be conducted to determine whether recruitment can be resumed, whether the protocol should be modified, or whether the study will be discontinued permanently. The FDA and reviewing IRB must be notified of any event that triggers suspension of enrollment in this study. If enrollment is suspended for safety reasons and it is deemed appropriate to resume the study, approval from the FDA and IRB must be obtained prior to resuming the study.

Regardless of whether recruitment is continued or not, all subjects injected with LUM015 or who were imaged with the LUM device at the time stopping rules were met will continue to be followed for safety.

In addition to the safety stopping rules outlined above, Lumicell may suspend or terminate this study at any time. The reasons for temporarily suspending or terminating the study may include but are not limited to the following:

- Subject enrollment is unsatisfactory

This document is confidential. Do not disclose or use except as authorized.

- Non-compliance that might significantly jeopardize the validity or integrity of the study.
- Lumicell decision to terminate development.

9 LUM015 FORMULATION AND ADMINISTRATION

9.1 Description

LUM015 has an abbreviated chemical formula of QSY21-Ahx-GGRK(Cy5)-AEEAc-C(mPEG20,000), where QSY21 is a fluorescence quencher from Life Technologies, Ahx is aminocaproic acid, G is the amino acid glycine, R is the amino acid arginine, K(Cy5) is the amino acid lysine conjugated with the fluorescent dye Cy5 (GE Healthcare), AEEAc is amino-ethoxy-ethoxy-acetyl, C is the amino acid cysteine and mPEG20,000 is a chain of methoxy-polyethylene glycol with an average molecular weight of 20,000 g/mol. The appearance of LUM015 is blue. The molecular weight of LUM015 is approximately 22,000 g/mol.

9.2 Form

LUM015 is supplied in 3-mL vials in powder form containing approximately 10 mg of LUM015, 10 mg of mannitol, 0.83 mg of sodium phosphate monobasic and 0.43 mg of sodium phosphate dibasic. LUM015 is provided by Lumicell, Inc.

9.3 Storage and stability

LUM015 must be stored at a temperature less than or equal to -20 °C (freezer) in powder form in the dark. Under these conditions, LUM015 is expected to be stable for up to 5 years. After reconstitution, LUM015 should be administered to the patient within 4 hours when stored at room temperature or 24 hours when stored refrigerated between 2°C and 8°C. Temperature logs are required for the storage freezers and refrigerators at the site's pharmacy.

9.4 Compatibility

Prior to injection, LUM015 should be reconstituted with 1.0 mL of 0.45% saline without glucose.

9.5 Handling

LUM015 should only be handled by qualified personnel familiar with procedures for reconstituting and injecting drugs or agents. Exposure to direct light for more than 5 minutes should be avoided.

9.6 Availability

LUM015 is an investigational agent and will be supplied free-of-charge from Lumicell, Inc.

9.7 Preparation

LUM015 is provided in amber vials in lyophilized form. Nominally each vial contains 10 mg of LUM015, 10 mg of mannitol, 0.83 mg of sodium phosphate monobasic and 0.43 mg of sodium phosphate dibasic. LUM015 will be reconstituted by adding 1.0 mL of 0.45% saline without glucose. Detailed preparation of the injection dose is explained in Appendix A. LUM015 from a single lot should be reconstituted for a given patient. Upon reconstitution, the pH of the solution is 6.5 and the osmolarity is 270 m-osm/L.

LUMICELL CONFIDENTIAL

This document is confidential. Do not disclose or use except as authorized.

Any reconstituted drug left in the vial that is not used for injection should be stored at 2-8 degrees C until it is confirmed that the patient did not exhibit an allergic reaction to the injection of LUM015. If an allergic reaction is reported, the remaining drug should be stored as -20 degrees C until a Lumicell representative notifies your site of how to proceed with the vial.

9.8 Administration

LUM015 is administered as a single dose of 1.0 mg/kg via peripheral intravenous (IV) injection in 0.45% saline without glucose between 2 and 6 hours prior to surgery. The IV line must be flushed with 10-20 mL of saline just prior to injection of LUM015 and the injection is immediately followed by a saline flush of 10-20 mL. If extravasation is suspected or confirmed, the injection must be stopped immediately and reported to Lumicell.

Per Principal Investigator's discretion, in order to protect subjects from potential anaphylactic reaction, prophylactic treatment with diphenhydramine and/or steroids may be administered.

In case of an allergic reaction to LUM015, sites should not discard any residual drug remaining in the used syringe. The syringe/used drug should be stored at -20 degrees C until a Lumicell representative notifies your site of how to proceed with the used syringe.

9.9 Ordering

LUM015 will be provided by Lumicell in enough quantities to cover the anticipated number of patients at each site. When more supply of LUM015 is needed, authorized pharmacy staff will notify the Principal Investigator and Lumicell. Lumicell will then supply the required vials of LUM015 for completing the study.

Kate Smith at Lumicell shall be notified via email at kate@lumicell.com for ordering LUM015.

9.10 Accountability

The investigator, or a responsible party designated by the investigator, must maintain a careful record of the inventory and disposition of LUM015 and the imaging devices utilizing appropriate forms.

9.11 Receipt and return of LUM015 and the LUM Imaging Devices

A drug inventory will be maintained by the clinical site's pharmacy. The inventory will include details of the LUM015 received and a clear record of when dispensed and for which subjects. This inventory record shall indicate the quantity and disposition of all investigational materials on hand at any time during the study.

LUM Imaging Devices will be delivered and installed by Lumicell personnel and Instructions for Use (IFU) will be provided. Lumicell personnel will also train the clinical staff on using the LUM Imaging System. Each clinical site investigator will maintain a device accountability record to document receipt and return of the LUM Imaging Devices.

At the end of the study, unused supplies of LUM015 and the investigational devices must be returned to Lumicell. A Lumicell representative will advise you when to return the supplies, as well as the proper shipping methods. Any LUM015 destroyed according to institutional policies will be documented in the Drug Accountability Record Form.

LUMICELL CONFIDENTIAL

This document is confidential. Do not disclose or use except as authorized.

This document is confidential. Do not disclose or use except as authorized.

10 STUDY CALENDAR

Baseline evaluations are to be conducted within 8 weeks (± 3 days) prior to subject enrollment (Day 1). All baseline assessments must be performed prior to administration of any study agent. The study calendar below applies to all subjects injected with LUM015 including those that do not undergo imaging due to any reason, including a failure in the LUM Imaging Device initialization.

Task	Baseline evaluation/screening	Day 1 / Enrollment	~2-14 days after surgery	Routine follow up visit
LUM015 administration		X		
Informed consent	X			
History	X			
Concurrent meds	X			
Physical exam (Ht, Wt, VS)	X			
Surgery/intraoperative imaging		X		
Histology margin assessment			X	
EKG	X			
CBC	X			X
Serum chemistry [a]	X			X
Adverse event/adverse device effect evaluation		X		X
Tumor measurements	X		X	
Radiologic evaluation	X			
B-HCG	X[b]			

a: Albumin, alkaline phosphatase, total bilirubin, BUN, calcium, chloride, glucose, potassium, total protein, SGOT [AST], SGPT [ALT], sodium.

b: Serum pregnancy test (women of childbearing potential).

11 SAFETY

A Phase 1 IND study in sarcoma and breast cancer patients was completed at Duke University Medical Center. The primary endpoint of this study was to evaluate the safety of the LUM015 drug component. As a secondary endpoint, the signals from normal and tumor tissue were measured ex vivo using the LUM Imaging Device component. Twelve (12) sarcoma patients and three (3) breast cancer patients (invasive ductal carcinoma) were injected with LUM015 at doses ranging from 0.5 mg/kg to 1.5 mg/kg with no adverse pharmacological activity reported. The only noticeable effect in the study subjects has been the blue/green discoloration of urine, which resolved within 12-24 hours post injection for most patients.

Currently, 60 breast cancer patients have been enrolled in the first two phases of the Feasibility Study (n=15 in Phase A, n=45 in Phase B), with 55 patients injected with LUM015. One patient enrolled in Phase B experienced blue discoloration at the injection site due to extravasation of LUM015 resulting from incorrect positioning of the intravenous catheter. After 92 days from injection, the blue discoloration disappeared but slight pallor of the skin at the injection site

This document is confidential. Do not disclose or use except as authorized.

remained. One patient enrolled (out of a total of 4) in the Phase C study experienced an anaphylactic reaction to the LUM015 injection. The patient recovered in <24 hours.

11.1 LUM015 pharmacokinetics

During the Phase 1 IND study, the first 3 eligible subjects were dosed at 0.5 mg/kg LUM015 and the second 3 subjects were dosed at 1.0 mg/kg LUM015, with both groups injected ~29 hours prior to surgery. Resected tissue was imaged with the LUM Imaging Device in the pathology suite. A comparison of pharmacokinetic data from the initial 6 subjects to preclinical mouse studies suggested that a higher tumor:normal signal ratio would be achieved if tissue was imaged 4-6 hours after injection. The next 3 subjects were injected with 1.0 mg/kg of LUM015 and followed by 3 subjects injected with 1.5 mg/kg of LUM015 and the final cohort of 3 subjects was injected with 0.5 mg/kg. These nine subjects were followed by surgical resection at approximately 6 hours after LUM015 injection (i.e. same day injection and surgery).

To evaluate the pharmacokinetic parameters of LUM015, plasma PK samples were collected at the following time-points: pre-dose, 10, 20 minutes, 1, 2, 4, 8, 12, 18, 22, and 48 hours post-dose. The 48-hour collection was optional. Analysis of LUM015 (as Frag 1 after trypsin digestion of plasma) and Fragment 1 (non-digested plasma) were measured by LC-MS/MS. Additional metabolites, Fragment 2 and Fragment 3 were measured by HPLC-fluorescence.

A non-compartmental approach within Win-Nonlin software was used for PK parameter estimation. Due to a relative complexity of the PK profile observed and data constraints inherent in the study (small number of patients per dose level, optional data from 22-48 hours), a compartmental modeling approach was not utilized. Out of the 15 subjects, the 48-hour time-point sample was collected in 9 patients. Thus, in order to make use of the wealth of data collected up to 22 hours (all 15 patients from the study), but also use potentially important 48-hour data from the limited set of 9 subjects, PK calculations were performed on both sets (22-hour and 48-hour) independently. A summary of the resulting PK parameters for LUM015 are presented in Table 2.

Table 2: Pharmacokinetic parameters for LUM015. SD = standard deviation.

PK Parameter	0.5 mg/kg	SD	1.0 mg/kg	SD	1.5 mg/kg	SD
T_{max} [hr]	0.11	0.17	0.06	0.13	0.00	0.00
C_{max} [ug/mL]	15.46	2.26	28.41	6.55	45.76	3.78
AUC_{last} [ug mL-1 h]	78.36	12.91	159.65	45.94	252.08	57.98
$T_{1/2}$ [h]	5.21	1.33	5.08	0.86	5.42	0.67

The pharmacokinetic data support rapid clearance of LUM015. After 22 and 48 hours, only 5% and 2% of the parent drug remain in plasma, respectively. The PK data support a multi-phased profile with linear pharmacokinetics.

11.2 Expected adverse events (associated with LUM015)

During Phase C of the Feasibility Study, one patient had an anaphylactic reaction to the LUM015 injection. This patient had a history of allergic reactions to contrast agents. The event was resolved within 24 hours of occurrence. We now consider anaphylactic reaction an expected adverse event,

LUMICELL CONFIDENTIAL

This document is confidential. Do not disclose or use except as authorized.

with probable occurrence and serious severity. Table 3 below lists the expected adverse events due to LUM015 injection with their estimated occurrence and severity.

During the IDE Feasibility Study in breast cancer, one subject experienced blue discoloration of the skin at the injection site due to extravasation of LUM015 resulting from incorrect positioning of the intravenous catheter. The blue discoloration faded over time, completely disappearing 92 days after the injection. Reduced pigment of the skin at the injection site was then noted. There were no other clinical sequelae identified in this patient. Extravasation and/or blue discoloration at the injection site has not been noted when the injection procedure was performed through a correctly positioned intravenous catheter.

Table 3: List of expected adverse events due to LUM015 administration.

**Adverse Events (Drug Related):**	**Occurrence:**	**Severity:**
LUM015 extravasation	Occasional	Minor
Chromaturia	Frequent	Negligible
Anaphylaxis/hypersensitivity	Probable	Serious

No adverse events related to the administration of LUM015 were observed during the Phase 1 IND safety study. Temporary blue/green discoloration of the urine was noted in these patients due to the blue color of LUM015. Prior to the Phase 1 study, LUM015 had not previously been used in humans. Our preclinical studies demonstrated that it was reasonably safe to proceed with a Phase 1 IND study in humans. Preclinical studies in rats showed no LUM015 related effects on clinical observations, FOB evaluation, body weights, food consumption, ocular condition, clinical chemistry, hematology and coagulation parameters or organ weight at doses up to 53-fold higher than in humans.

The results from the repeat dose toxicity study in dogs (performed by NCI) show that administration of 0.5 or 10.0 mg/kg of LUM015 intravenously once daily for seven consecutive days (8 total doses) did not cause any observable target organ toxicities. The only observable effect was hypersensitivity in several dogs.

The results from a repeat dose toxicity study in rabbits show that administration of 15 mg/kg of LUM015 intravenously one daily for seven consecutive days was well tolerated. The test article-related findings were limited to transient blue/green discoloration of the urine. There were no test article-related adverse events observed and no hypersensitivity seen.

Clinicians should be prepared for a possible allergic reaction to occur during each administration of LUM015. Standing orders should be in place in the event of an allergic reaction for immediate intervention including administration of diphenhydramine, prednisone or both. If a study subject develops a Grade 3 (or greater) allergic reaction as defined in CTCEA 5.0, Lumicell should be contacted within 24 hours of the event:

LUMICELL CONFIDENTIAL

This document is confidential. Do not disclose or use except as authorized.

To Lumicell:
Felix Geissler, MD, PhD
Phone: 609-498-4809
Email: felix@lumicell.com
fax: 617-507-5512

11.3 Expected adverse device effects (associated with the LUM Imaging System)

To date, 64 patients (15 in Phase A, 45 in Phase B, and 4 patients to date in Phase C) have been imaged intraoperatively with the LUM Imaging System with no observable adverse device effects (the one incident of anaphylaxis in Phase C was considered an SAE but not an adverse device effect). Based on observation to date, there are no known anticipated or expected adverse device effects.

12 ADVERSE EVENT REPORTING REQUIREMENTS

12.1 Definitions

12.1.1 Adverse Event (AE)

An adverse event (AE) is any undesirable sign, symptom or medical condition or experience that develops or worsens in severity after starting the first dose of study treatment or any procedure specified in the protocol, even if the event is not considered to be related to the study. The severity of any possible AE observed will be classified per the grading established by the Common Terminology Criteria for Adverse Events (CTCAE) 5.0.

Abnormal laboratory values or diagnostic test results constitute adverse events only if they induce clinical signs or symptoms or require treatment or further diagnostic tests.

12.1.2 Serious adverse event (SAE)

A serious adverse event (SAE) is any adverse event, occurring at any dose and regardless of causality that:

- Results in death
- Is life-threatening. Life-threatening means that the person was at immediate risk of death from the reaction as it occurred, i.e., it does not include a reaction which hypothetically might have caused death had it occurred in a more severe form.
- Requires or prolongs inpatient hospitalization (i.e., the event required at least a 24-hour hospitalization or prolonged a hospitalization beyond the expected length of stay). Hospitalization admissions and/or surgical operations scheduled to occur during the study period but planned prior to study entry are not considered SAEs if the illness or disease existed before the person was enrolled in the trial, provided that it did not deteriorate in an unexpected manner during the trial (e.g., surgery performed earlier than planned).
- Results in persistent or significant disability/incapacity. Disability is defined as a substantial disruption of a person’s ability to conduct normal life functions.
- Is a congenital anomaly or birth defect; or

This document is confidential. Do not disclose or use except as authorized.

- Is an important medical event when, based upon appropriate medical judgment, it may jeopardize the subject and require medical or surgical intervention to prevent one of the outcomes listed above. Examples of such medical events include allergic bronchospasm requiring intensive treatment in an emergency room or at home; blood dyscrasias or convulsions that do not result in inpatient hospitalization, or the development of drug dependency or drug abuse.

Events **not** considered to be serious adverse events are hospitalizations for:

- routine treatment or monitoring of the studied indication, not associated with any deterioration in condition, or for elective procedures
- elective or pre-planned treatment for a pre-existing condition that did not worsen
- respite care

12.1.3 Adverse device effect (ADE)

An adverse device effect (ADE) is an adverse event which is at least possibly related to the device. ADEs are not considered serious adverse events.

12.1.4 Unanticipated adverse device effects (UADEs)

Unanticipated adverse device effect (UADE) is any serious adverse effect on health or safety or any life-threatening problem or death cause by or associated with, a device, if that effect, problem, or death was not previously identified in nature, severity, or degree of incidence in the investigation plan or application, or any other unanticipated serious problem associated with a device that relates to the rights, safety or welfare of subjects.

12.1.5 Expectedness

Adverse events (AEs) and adverse device effects (ADEs) can be 'Expected' or 'Unexpected'.

12.1.5.1 Expected adverse event or adverse device effect

Expected AEs and ADEs are those that have been previously identified as resulting from administration of the agent or use of the device. For the purposes of this study, an AE or ADE is considered expected when it appears in the current AE/ADE list, the Investigator's Brochure, the Instructions for Use or is included in the informed consent document as a potential risk.

Refer to Section 11.2 and 11.3 for expected adverse events associated with LUM015 and expected adverse device effects associated with the imaging device, respectively.

12.1.5.2 Unexpected adverse event or adverse device effect

For the purposes of this study, an AE or ADE is considered unexpected when it varies in nature, intensity or frequency from information provided in the current AE/ADE list, the Investigator's Brochure, the Instructions for Use, or when it is not included in the informed consent document as a potential risk.

LUMICELL CONFIDENTIAL

This document is confidential. Do not disclose or use except as authorized.

12.1.6 Attribution

Attribution is the relationship between an adverse event or serious adverse event and the study treatment. Attribution will be assigned as follows:

- Definite – The AE or ADE is clearly related to the study treatment.
- Probable – The AE or ADE is likely related to the study treatment.
- Possible – The AE or ADE may be related to the study treatment.
- Unlikely - The AE or ADE is doubtfully related to the study treatment.
- Unrelated - The AE or ADE is clearly NOT related to the study treatment.

12.2 **Procedures for recording and reporting safety**

The principal investigator will assess the occurrence of AEs, SAEs, ADEs and UADEs at all subject evaluation time points during the study.

All AEs, SAEs, ADEs and UADEs whether reported by the subject, discovered during questioning, directly observed, or detected by physical examination, laboratory test or other means, will be recorded in the subject's medical record and on the appropriate study-specific case report forms.

For events related to the imaging agent, the descriptions and grading scales found in the revised NCI Common Terminology Criteria for Adverse Events (CTCAE) version 5.0 will be utilized for AE reporting. All appropriate treatment areas should have access to a copy of the CTCAE version 5.0.

A copy of the CTCAE version 5.0 can be downloaded from the CTEP website at:

https://ctep.cancer.gov/protocolDevelopment/electronic_applications/ctc.htm

12.3 **Reporting requirements**

The study must be conducted in compliance with FDA regulations, local safety reporting requirements, and reporting requirements of the principal investigator and/or IRB.

The Investigator will be responsible to report SAEs that occur at the Institution to the IRB in accordance with IRB requirements. It is the responsibility of the principal investigator to report serious adverse events to Lumicell and/or others as described below.

12.4 **Reporting to Lumicell**

12.4.1 Serious Adverse Event Reporting

All serious adverse events that occur after the initial dose of LUM015 Imaging Agent must be reported to Lumicell using the provided SAE form. This includes events meeting the following criteria:

- Grade 2 (moderate) and Grade 3 (severe) Events – Only events that are unexpected and possibly, probably or definitely related/associated with the intervention (with the exception of an allergic reactions, which will also be included as SAEs).
- All Grade 4 (life-threatening or disabling) Events – Unless expected AND specifically listed in the protocol as not requiring reporting.

LUMICELL CONFIDENTIAL

This document is confidential. Do not disclose or use except as authorized.

- All Grade 5 (fatal) Events – When the subject is enrolled and actively participating in the trial OR when the event occurs within 30 days of the last study intervention.

  Note: If the subject is in long term follow up, report the death at the time of continuing review.

The principal investigator must report each serious adverse event to Lumicell within 24 hours of learning of the occurrence. In the event that the Investigator does not become aware of the serious adverse event immediately (e.g., subject sought treatment elsewhere), the principal investigator is to report the event within 24 hours after learning of it and document the time of his or her first awareness of the adverse event. Report serious adverse events by telephone, email or facsimile to:

To Lumicell:
Felix Geissler, MD, PhD
Phone: 609-498-4809
Email: felix@lumicell.com
fax: 617-507-5512

12.4.2 Unanticipated adverse device effects

All unanticipated adverse device effects (UADEs) that occur during the study are considered serious and must be reported to Lumicell using the provided SAE form.

The principal investigator must report each unanticipated adverse device effect to Lumicell within 24 hours of learning of the occurrence. In the event that the Investigator does not become aware of the unanticipated adverse device effect immediately (e.g., subject sought treatment elsewhere), the principal investigator is to report the event within 24 hours after learning of it and document the time of his or her first awareness of the adverse device effect. Report unanticipated adverse device effects by telephone, email or facsimile to:

To Lumicell:
Felix Geissler, MD, PhD
Phone: 609-498-4809
Email: felix@lumicell.com
fax: 617-507-5512

12.4.3 Non-serious adverse event and adverse device effect reporting

Non-serious adverse events and non-serious adverse device effects will be reported to Lumicell on the adverse events Case Report Forms.

12.5 Reporting to the Institutional Review Board (IRB)

The investigative site will report adverse events, serious adverse events, adverse device effects and unexpected adverse device effects directly to the IRB in accordance with IRB requirements.

12.6 Reporting to the Food and Drug Administration (FDA)

Lumicell or its agents will report to the FDA via their IDE as required in 21 CFR Parts 312 & 812 and as additionally described in this protocol.

LUMICELL CONFIDENTIAL

This document is confidential. Do not disclose or use except as authorized.

12.7 Reporting to hospital risk management

The principal investigator will report to their local Risk Management office any subject safety reports or sentinel events that require reporting according to institutional policy.

12.8 Monitoring of adverse events/adverse device effects and period of observation

All adverse events and adverse device effects, both serious and non-serious, and deaths that are encountered from initiation of study intervention, throughout the study, and within the first post-operative follow-up visit should be followed to their resolution, or until the principal investigator assesses them as stable, or the principal investigator determines the event to be irreversible, or the subject is lost to follow-up. The presence and resolution of AEs, ADEs, SAEs and UADEs (with dates) should be documented on the appropriate case report form and recorded in the subject's medical record to facilitate source data verification.

For some SAEs/UADEs, the investigator or designee may follow-up by telephone, fax, and/or monitoring visit to obtain additional case details deemed necessary to appropriately evaluate the SAE/UADE report (e.g., hospital discharge summary, consultant report, or autopsy report).

Subjects should be instructed to report any serious post-study event(s) that might reasonably be related to participation in this study. The principal investigator should notify Lumicell and the IRB of any unanticipated death or adverse event occurring after a subject has discontinued or terminated study participation that may reasonably be related to the study.

12.9 Protocol deviations

Protocol deviations to ongoing studies are any unapproved changes in the study design and/or procedures that are within the Investigator's control and not in accordance with the IRB approved protocol. The Principal Investigator will complete the Protocol Deviation form provided in the Study Binder. Additionally, the reviewing IRB has to be contacted (if applicable per the IRB's requirements) by the Investigator if a Protocol Deviation might either affect the participant's right, safety or well-being, or might significantly affect the completeness, accuracy and reliability of the data. The Protocol Deviation form provided by Lumicell will be used for this purpose.

Note: The Principal Investigator will summarize any protocol deviations noted during the course of the study in a final statement.

In the event that the deviation was identified by the Investigator, the Investigator may use the Lumicell deviation form to summarize and assess the impact of the deviation. Similarly, the Lumicell protocol deviation form may be utilized to communicate a deviation that is identified by Lumicell to the investigator. In both cases, the Investigator must provide Lumicell with a copy of the form submitted to their IRB or use the Lumicell form to document the deviation. The deviation will be categorized in the description section of the form as a Protocol Waiver or Protocol Deviation.

Examples of major Protocol Deviations include, but are not limited to the following:

- Inadequate or nonexistent informed consent
- Unreported Adverse Events/Adverse Device Effects

LUMICELL CONFIDENTIAL

This document is confidential. Do not disclose or use except as authorized.

- Data Corruption or falsified data

13 DATA AND SAFETY MONITORING

13.1 **Data reporting**

13.1.1 Method

Lumicell will collect, manage, and monitor data for this study.

13.1.2 Data submission

It is the expectation that all case report forms (CRFs) will be completed in a contemporaneous manner at the time of study related procedures. Timely completion of the CRFs will allow Lumicell to monitor the study conduct and data in an effective manner.

13.2 **Safety review**

For this study, an independent safety reviewer (Medical Safety Monitor) will review and monitor adverse events data from this trial on an ongoing basis. The Medical Safety Monitor will be a clinical specialist with experience in oncology and who has no direct relationship with the study. Information that raises any questions about subject safety will be addressed with the Principal Investigator and study team.

13.3 **Monitoring**

Lumicell's monitoring process will be initiated with the initial visits to sites to assure that they meet the qualification requirements and have adequate resources and experience to conduct the study protocol. When the site has met all qualification criteria, submitted all initial regulatory documentation, and received IRB approval for the study, Lumicell will conduct a study initiation visit (either face to face, or via web) in which the site team will receive protocol specific training in addition to a review of all investigator responsibilities, and expectations of Lumicell. Throughout the conduct of the study Lumicell will conduct intermittent site visits to assure compliance with the protocol and all applicable regulations. During these visits, there will be a source data verification of critical data points transcribed to the CRF. At study completion, Lumicell will conduct a study closure visit. This study visit will assure that all site regulatory documentation is present and updated; all data queries are resolved; investigator sign-off/approval of all applicable study data submitted to Lumicell has occurred; and all final disposition instructions from Lumicell have been delivered to the site investigator.

Involvement in this study as a participating investigator implies acceptance of the approved protocol, the potential for audits or inspections, including source data verification, by representatives of the FDA, Lumicell (or their affiliates), the institutional review board, or their representatives. The purpose of these audits or inspections is to examine study-related activities and documents to determine whether these activities were conducted and data were recorded, analyzed, and accurately reported in accordance with the protocol, institutional policy, Good Clinical Practices (GCPs), and any applicable regulatory requirements.

LUMICELL CONFIDENTIAL

This document is confidential. Do not disclose or use except as authorized.

All data will be monitored for timeliness of submission, completeness, accuracy, and adherence to protocol requirements. Monitoring will begin at the time of subject registration and will continue during protocol performance and completion.

13.4 Data Safety Monitoring Board

An independent Data Safety Monitoring Board (DSMB) will be established to review the safety of the LUM Imaging System and to review any SAEs that occur during the study. The DSMB will develop and follow a DSMB Charter. The DSMB will be composed of at least 2 oncology specialists (at least one of whom specializes in surgical oncology) and a statistician. The DSMB will be provided with all reports of adverse events including SAEs regardless of investigator causality assessments.

Following the initial meeting, DSMB meetings will occur on a periodic basis in accordance with the DSMB charter. The chairperson of the DSMB will also be immediately provided with the report of any SAE that is judged as possibly, probably, or definitely attributable to treatment with the investigational product. The charter of the DSMB will specify that this committee is charged with providing periodic reports to Lumicell that contain recommendations that include, but are not limited to, (a) continuation of the study, and (b) termination of the study.

14 REGULATORY CONSIDERATIONS

14.1 Protocol review and amendments

This protocol, the proposed informed consent and all forms of subject information related to the study (e.g., advertisements used to recruit subjects) and any other necessary documents must be submitted, reviewed and approved by a properly constituted IRB governing each study location.

Any changes made to the protocol must be mutually agreed upon by Lumicell. Such changes must be submitted as amendments and must be approved by the IRB prior to implementation. Any changes in study conduct must be reported to the IRB. Lumicell will disseminate protocol amendment information to all participating investigators.

All decisions of the IRB concerning the conduct of the study must be made in writing.

In addition, any modifications to the protocol, consent or case report forms will be submitted to the FDA.

14.2 Informed consent

All subjects must be provided a consent form describing this study and providing sufficient information for subjects to make an informed decision about their participation in this study. The formal consent of a subject, using the current IRB approved consent form, must be obtained before any study-related procedures are performed. The consent form must be signed and dated by the subject or the subject's legally authorized representative, and by the person obtaining the consent. The subject must be given a copy of the signed and dated consent document. The original signed copy of the consent document must be retained in the medical record or research file.

14.3 Ethics and Good Clinical Practice (GCP)

LUMICELL CONFIDENTIAL

This document is confidential. Do not disclose or use except as authorized.

This study is to be conducted according to the following considerations, which represent good and sound research practice:

- E6 Good Clinical Practice: Consolidated Guidance
- US Code of Federal Regulations (CFR) governing clinical study conduct and ethical principles that have their origin in the Declaration of Helsinki
    - Title 21 Part 11 – Electronic Records; Electronic Signatures
    - Title 21 Part 50 – Protection of Human Subjects
    - Title 21 Part 54 – Financial Disclosure by Clinical Investigators
    - Title 21 Part 56 – Institutional Review Boards
    - Title 21 Part 812 – Investigational Device Exception Application
- State laws
- Institutional research policies and procedures
- Contractual agreements

It is understood that deviations from the protocol should be avoided, except when necessary to eliminate an immediate hazard to a research subject. In such case, the deviation must be reported to the IRB according to the local reporting policy.

14.4 **Study documentation**

The investigator and all designees must prepare and maintain adequate and accurate case histories designed to record all observations and other data pertinent to the study for each research subject. This information enables the study to be fully documented and the study data to be subsequently verified.

Original source documents supporting entries in the case report forms include but are not limited to hospital records, clinical charts, laboratory and pharmacy records, recorded data from automated instruments, microfiches, photographic negatives, microfilm or magnetic media, and/or x-rays.

14.4.1 Study documentation practices

a) The investigator must maintain adequate records to enable the conduct of the study to be fully documented
b) All records, including electronic forms, must be filled out completely. Complete each space or blank. If there is no information to go into a space or blank, then use the symbol "N/A". If an entire section or page of a record is "N/A", it is acceptable to indicate this by drawing one line through the entire section/page and use "N/A" near that line. It is acceptable to check a box marked "N/A" to indicate that a section or an entire page is not applicable. The use of 'White-Out' or similar correction fluid is forbidden.
c) Handwritten dates are always to be recorded in the sequence of the month, day and year (mm/dd/yy or yyyy) unless otherwise specified.
d) Use of highlighters on records is acceptable so long as the highlighter color does not obscure the underlying text if the record is copied.
e) Accuracy is required. Always verify entries are correct and consistent with other information.
f) Signatures are to be authentic.

This document is confidential. Do not disclose or use except as authorized.

g) Recorded dates are to be the actual dates in which the activities were recorded. Back-dating is forbidden.
h) If drinks or chemicals are spilled on original records, dry them off to the best of your ability, make an immediate photocopy and make a notation of the event on the copy. Retain the original record except in the event of contamination with a hazardous material.
i) All forms must be filled out using non-erasable pen and must be legible. The use of blue or black ink is preferred.
j) Errors must be crossed out with a single line, the correction inserted, and the change initialed and dated by the approved person making the correction. The reason for the correction must be stated.

14.5 Records retention

All study-related documents must be retained for the maximum period required by applicable federal regulations and guidelines, institutional policies or contractual agreement between Lumicell and the participating institution.

15 STATISTICAL CONSIDERATIONS

The objectives for expanding the Feasibility Study into Phase C at multiple sites are to:

- Collect data to refine and verify the tumor detection algorithm
- Complete hands-on training of the surgeons and clinical staff that will be participating in the pivotal study to evaluate the efficacy and safety of the LUM Imaging System in breast cancer surgeries
- Identify and address any site-specific or user-specific issues for using the LUM Imaging System in breast cancer surgeries
- Collect safety and efficacy data

15.1 General methods

In addition to documenting the training status of the clinical staff on the LUM Imaging System, data from this study will be used to continue refining the tumor detection algorithm. For categorical variables, summary tabulations of the number and percentage of patients within each category (with a category for missing data) of the parameter will be presented. For continuous variables, the number of patients, mean, median, standard deviation, minimum, and maximum values will be presented.

Each data value for all the included patients will be presented using patient data listings, sorted by patient number, unless otherwise specified.

All data summary tabulations will be presented by study site and for the overall study population, as appropriate.

15.1.1 Disposition of subjects

LUMICELL CONFIDENTIAL

This document is confidential. Do not disclose or use except as authorized.

Demographic and baseline characteristics data will be summarized for all patients. The total number of patients screened, enrolled, completed and reason for termination will also be summarized.

15.2 Sample size

Analyses during the Phase C study may lead to a change of algorithm (i.e., refinement of the threshold). The tumor detection algorithm will be locked in place before the start of the pivotal study based on the results of this Phase C study. The tumor detection algorithm lock will be contingent on the accrual of enough "pathology positives" to yield a ~7% margin of error in sensitivity. "Pathology positive" is defined as an imaged surface that contains cancer as identified by histopathology. A single patient may give more than one "pathology positive" image. Given that 19 pathology positive samples were acquired during the Phase B study, an additional 32 pathology positives will be needed to achieve the targeted margin of error. Because 40 patients in the Phase B study yielded 19 pathology positives, we expect to image approximately 67 patients during the training portion of the pivotal study (Phase C) before locking the threshold algorithm in place for the pivotal study. However, because the incidence of pathology positives is largely unknown at the sites participating in the future pivotal trial, the study will continue until enough patients have been enrolled to collect the data to lock the tumor detection algorithm. This number of patients will not exceed 250 patients.

Based on our experience from Phase A and Phase B of the feasibility study, we anticipate that with up to 5 patients per surgeon and approximately 1-3 surgeons per site, we may identify and address any site-specific and surgeon-specific issues related to the use of the LUM Imaging System. We anticipate expanding the study to a maximum of 20 sites; thus, we expect to have up to 250 patients in Phase C.

If tumor detection algorithm verification is complete, yet not all surgeons and clinical staff have completed training on the LUM Imaging System, these institutions can continue the training process as a lead-in to any Lumicell Imaging Studies they may participate in the future.

15.3 Populations for analysis

Safety evaluations will be based on all patients who enter the study that are injected with LUM015, regardless on whether the LUM Imaging Device was used for intraoperative imaging (there may be subjects injected with LUM015 and not imaged intraoperatively for a variety of reasons). Subjects imaged with the LUM Imaging System will be included in the analysis of the imaging data unless otherwise specified.

15.4 Exclusions

Subjects injected with LUM015 but not imaged with the LUM Imaging Device will not be included in the analysis of the imaging data and will not count towards the 3 patients per surgeon to complete the training. They will be included in the safety analysis. Other subjects excluded from any analysis will be indicated with a rationale as to why the patient was excluded.

15.5 Efficacy and intraoperative imaging data analysis

LUMICELL CONFIDENTIAL

This document is confidential. Do not disclose or use except as authorized.

The tumor detection algorithm optimized during Phase B will be implemented in Phase C to guide the resection of therapeutic SCMs. The imaging data will be compared against histopathology analysis of resected tissue on the side of the tissue where in vivo imaging took place. The data obtained from the different surgeons at each site will be used to further train the tumor detection algorithm. The combined algorithm training data from Phase B and Phase C will be used to estimate the expected results in the pivotal trial to properly power that study.

15.6 Safety analysis

Adverse events and adverse device effects will be collected, analyzed and tabulated as needed. Serious adverse events (SAE) and unanticipated adverse device effects (UADEs) will be tabulated separately and analyzed with respect to their severity.

16 PUBLICATION PLAN

This study has been registered at clinicaltrials.gov. The results of this study will be made public within 12 months of the completion of the study.

Reference the Clinical Trial Agreement for detailed publication policy information.

17 REFERENCES

1. Chow, S.C., J. Shao, and H. Wang, *Sample Size Calculations in Clinical Research*. 2003, New York: Marcel Dekker, Inc.
2. Weissleder, R. and U. Mahmood, *Molecular imaging.* Radiology, 2001. **219**(2): p. 316-33.
3. Mohamed, M.M. and B.F. Sloane, *Cysteine cathepsins: multifunctional enzymes in cancer.* Nat Rev Cancer, 2006. **6**(10): p. 764-75.
4. Gocheva, V. and J.A. Joyce, *Cysteine cathepsins and the cutting edge of cancer invasion.* Cell Cycle, 2007. **6**(1): p. 60-4.
5. Joyce, J.A., et al., *Cathepsin cysteine proteases are effectors of invasive growth and angiogenesis during multistage tumorigenesis.* Cancer Cell, 2004. **5**(5): p. 443-53.
6. Gocheva, V., et al., *IL-4 induces cathepsin protease activity in tumor-associated macrophages to promote cancer growth and invasion.* Genes Dev, 2010. **24**(3): p. 241-55.
7. Jaffer, F.A., et al., *Optical visualization of cathepsin K activity in atherosclerosis with a novel, protease-activatable fluorescence sensor.* Circulation, 2007. **115**(17): p. 2292-8.
8. Chen, B. and M.O. Platt, *Multiplex Zymography Captures Stage-specific Activity Profiles of Cathepsins K, L, and S in Human Breast, Lung, and Cervical Cancer*. J Transl Med, 2011. **9**: p. 109.
9. Lecaille, F., J. Kaleta, and D. Bromme, *Human and parasitic papain-like cysteine proteases: their role in physiology and pathology and recent developments in inhibitor design.* Chem Rev, 2002. **102**(12): p. 4459-88.
10. Naidu, R., et al., *Expression of c-erbB3 protein in primary breast carcinomas.* Br J Cancer, 1998. **78**(10): p. 1385-90.
11. Thomssen, C., et al., *Prognostic value of the cysteine proteases cathepsins B and cathepsin L in human breast cancer*. Clin Cancer Res, 1995. **1**(7): p. 741-6.
12. Lerebours, F., I. Bieche, and R. Lidereau, *Update on inflammatory breast cancer.* Breast Cancer Res, 2005. **7**(2): p. 52-8.

LUMICELL CONFIDENTIAL

This document is confidential. Do not disclose or use except as authorized.

13. Nouh, M.A., et al., *Cathepsin B: a potential prognostic marker for inflammatory breast cancer*. J Transl Med, 2011. **9**: p. 1.
14. Kreike, B., et al., *Continuing risk of ipsilateral breast relapse after breast-conserving therapy at long-term follow-up.* Int J Radiat Oncol Biol Phys, 2008. **71**(4): p. 1014-21.
15. Singletary, S.E., *Surgical margins in patients with early-stage breast cancer treated with breast conservation therapy*. Am J Surg, 2002. **184**(5): p. 383-93.
16. Smitt, M.C., et al., *Predictors of reexcision findings and recurrence after breast conservation.* Int J Radiat Oncol Biol Phys, 2003. **57**(4): p. 979-85.
17. Dillon, M.F., et al., *A pathologic assessment of adequate margin status in breast-conserving therapy.* Ann Surg Oncol, 2006. **13**(3): p. 333-9.
18. Kurniawan, E.D., et al., *Predictors of surgical margin status in breast-conserving surgery within a breast screening program.* Ann Surg Oncol, 2008. **15**(9): p. 2542-9.
19. Schiller, D.E., et al., *Factors associated with negative margins of lumpectomy specimen: potential use in selecting patients for intraoperative radiotherapy.* Ann Surg Oncol, 2008. **15**(3): p. 833-42.
20. Smitt, M.C. and K. Horst, *Association of clinical and pathologic variables with lumpectomy surgical margin status after preoperative diagnosis or excisional biopsy of invasive breast cancer.* Ann Surg Oncol, 2007. **14**(3): p. 1040-4.
21. Waljee, J.F., et al., *Predictors of re-excision among women undergoing breast-conserving surgery for cancer*. Ann Surg Oncol, 2008. **15**(5): p. 1297-303.
22. Coopey, S., et al., *The Safety of Multiple Re-excisions after Lumpectomy for Breast Cancer*. Ann Surg Oncol, 2011.
23. Coopey, S.B., et al., *Retrospective reveiw of breast cancer patients who underwent lumpectomy from 2004-2006.* Am J Surg, 2011: p. *in press*.

LUMICELL CONFIDENTIAL

This document is confidential. Do not disclose or use except as authorized.

18 APPENDICES

Appendix A: Protocol for preparation of LUM015 for injection

1 Purpose

The purpose of this protocol is to describe the dose preparation for the LUM015 Imaging Agent.

2 Materials

- LUM015 Formulated, stored frozen (-20°C)
- Sterile syringe
- 0.45% Saline without glucose

3 Methods

1) After reconstitution, storage of LUM015 is limited to 4 hours at room temperature or 24 hours when stored refrigerated between 2°C and 8°C.
2) Obtain the most recent accurate weight of the subject in kg.
   - Note: If weight is measured in lbs, multiply value by 0.45359 to obtain kg. Example: 155lbs=(155*0.45359)kg=70.306kg
3) Calculate total dose for subject according to protocol.
   - Example: If dose level is 1.0 mg/kg and weight is 70kg, then dose = 1.0*70=70 mg.
4) Obtain the required number of vials to administer the full dose. Each vial contains 10mg LUM015.
   - Example: If 70mg is the required dose, 70/10=7 vials.
   - Example 2: If the dose level is 1.0 mg/kg and the patient weight is 63 kg, the required number of vial for the dose of 63 mg is 63/10 = 6.3; thus 7 vials would be needed for this patient.
5) Allow bottles to acclimate to room temperature for 5 minutes.
6) Reconstitute each vial of LUM015 with 1mL of 0.45% saline without glucose.
7) Combine the calculated dose from the individual vials in one large syringe.
8) Ensure the vial is homogeneous in blue color (see Figure 3 below).
9) The IV line must be flushed with 10-20 mL of saline just prior to injection of LUM015 and the injection is immediately followed by a saline flush of 10-20 mL.
10) Inject slow bolus into the subject (over three minutes).

LUMICELL CONFIDENTIAL

This document is confidential. Do not disclose or use except as authorized.

11) In case of an allergic reaction to LUM015, sites should not discard any residual drug remaining in the used syringe or unused portion of reconstituted vials. This drug should be stored at -20 degrees C until a Lumicell representative notifies the site of how to proceed with the used syringe.

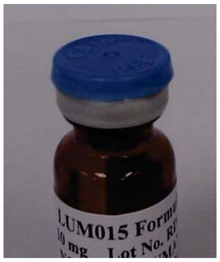

Figure 1: Vial with protective cap

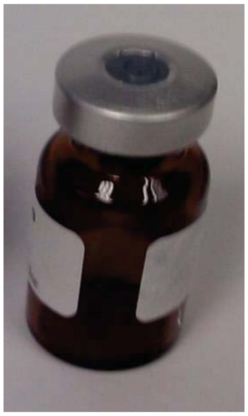

Figure 2: Empty vial with protective cap removed

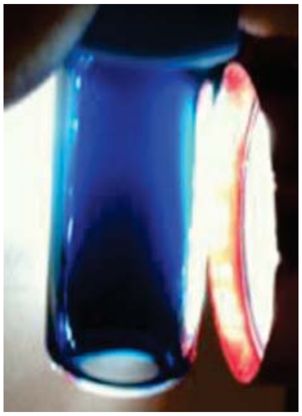

Figure 3: Dissolved compound

4 Caution

- **Ensure there are no air bubbles in the syringe prior to injection.**
- **Ensure there are no solid particles visible on the side walls of the syringe barrel prior to injection.**
- **Do not use compound if the date has exceeded the expiration date.**
- **Exposure to direct light for more than 5 minutes should be avoided.**

LUMICELL CONFIDENTIAL

This document is confidential. Do not disclose or use except as authorized.

Appendix A: Method for removing, orienting, inking and naming the main specimen and shaved cavity margins.

The methods described below are adapted from Cabioglu, et al. (ASO 14:1458-1471; 2007). This method applies to both palpable and non-palpable breast masses.

Procedure:

1. Surgeon performs surgery attempting to obtain grossly negative margins in the main specimen.
2. Surgeon places orientation stitches at the superior and lateral orientations of the main specimen before it is removed from the patient. **It is extremely important to maintain the orientation of the specimen relative to the lumpectomy cavity to be able to correlate LUM images of the cavity with pathology examination of the main specimen**.
3. Surgeon may elect to remove additional tissue based on palpation of the specimen or lumpectomy cavity walls, x-ray imaging of the main specimen (mainly for non-palpable masses) or visual inspection. If additional tissue is removed, the tissue must be labeled as "additional" and include the orientation from where it was removed (see
4. Appendix **B** for naming convention of resected tissue). For example, if the surgeon decides to remove additional tissue from the medial orientation, then that tissue should be named as "additional medial excision".
5. Surgeon inks all surfaces of the main specimen using a differently colored ink for each orientation with the kit procured from a third-party vendor by Lumicell. This kit is not part of the LUM Imaging System.
6. If additional tissue is removed, the surgeon should ink as many surfaces as possible maintaining the same color used for a given orientation in the main specimen. For example, if the superior orientation of the main specimen is inked yellow, then the margin side of a SCM from the superior orientation should be inked yellow.
7. All tissue is sectioned in the surgical pathology suite serially in 3-5 mm sections.
8. When therapeutic SCMs are removed, the margin side should be marked with a stitch. These therapeutic SCMs should be named as specified in
9. Appendix **B**. The opposite side from the stitch must be imaged with the LUM Imaging Device in the operating room. These shaves should be inked per institution's standard procedure.
10. When selective or comprehensive SCMs are removed, the margin side should be marked with a stitch. These SCMs they should be named as specified in
11. Appendix **B**. These shaves should be inked per institution's standard procedure.

LUMICELL CONFIDENTIAL

This document is confidential. Do not disclose or use except as authorized.

Appendix B: Guidelines for naming of resected tissue

To facilitate data collection and analysis standard naming convention must be used to label all the resected tissue at all sites. Table C1 below provides the guidelines for naming resected tissue. This naming convention must follow the resected tissue since its removal to the final pathology report.

Table C1: Guidelines for naming resected tissue.

Tissue	Naming convention	Example case	Naming example
Main specimen	Main specimen [side of breast]	Main specimen removed from the left breast	Main specimen, left breast
Additional tissue resected based on palpation, visual inspection and/or X-ray imaging of intact main specimen	Additional [orientation]	Additional tissue removed from superior due to palpation of main specimen	Additional superior
Therapeutic shaves	[Orientation] [sequential shave number for an orientation] Therapeutic	First therapeutic shave from medial orientation	Medial 1 Therapeutic
Standard of care shaves	[Orientation] 1 SOC	SOC shave from superior margin	Superior 1 SOC

LUMICELL CONFIDENTIAL

This document is confidential. Do not disclose or use except as authorized.

Appendix D: Site-specific sub-study to evaluate complement activation for LUM015 investigational agent

A select number of institutions will participate in a sub-study to evaluate complement activation of LUM015.

Rationale/Background:

LUM015 is a fluorescence-based imaging agent that accumulates within and around cells. LUM015 is administered in the main study to the patient via intravenous injection 4 ± 2 hours prior to surgery.

In order to understand the potential of LUM015 to induce a Type I hypersensitivity response in patients, changes in levels of three readily determined clinical lab measures (serum histamine, tryptase and complement) associated with a Type I response are to be evaluated in a cohort of patients that receive LUM015. As any such responses occur promptly after parentheral exposure to a hypersensitivity inducing moiety, vital signs and blood samples are to be collected prior to LUM015 injection and at 15, 30 and 60 minutes post injection.

Primary objective: Evaluate complement activation of LUM015 via analysis of vital signs, tryptase, histamine, and total complement levels in patients being injected with LUM015

Enrollment: 10-25 patients will be enrolled at 2-3 selected institutions.

Schedule of Events: Day 1*

**Histamine**	**Tryptase**	**Total Complement**	**Vital Signs**[a]
Pre-Dose	Pre-Dose	Pre-Dose	Pre-Dose
15 Minutes	15 Minutes	15 Minutes	15 Minutes
30 Minutes	30 Minutes	30 Minutes	30 Minutes
60 Minutes	60 Minutes	60 Minutes	60 Minutes

*All safety labs and vital signs should be collected prior to subjects going into surgery.
[a] Vital Signs should include Temperature and Blood Pressure

Review the Lab Manual for processing and shipping instructions of the samples to the central lab.

Statistical Analysis Plan

All numerical data (histamine, tryptase, total and differentiated complement) will be input into a formatted NCSS data base and then curated using graphical and numeric methods. Once the data sets have thus been verified, each variable will be analyzed for differences from baseline at each

This document is confidential. Do not disclose or use except as authorized.

of the time points by ANOVA followed by a suitable *post hoc* analysis. *P* values of 0.05 or less will serve as the criterion for difference being considered significant for endpoints.

Gad SC**.** (2005) *Statistics and Experimental Design for Toxicologists and Pharmacologists*, 4th Ed. CRC Press, Boca Raton, FL.

This document is confidential. Do not disclose or use except as authorized.

Appendix E: Confidentiality and Confirmation Signatures

Confidentiality agreement:

The Principal Investigator agrees to handle all information and documentation received from Lumicell, Inc. under the terms of the study agreement as well as the work performed and the results obtained during the duration and after termination of the agreement confidentially. Accordingly, all separate publications and lectures with any reference to the object of this agreement need the prior written consent of Lumicell, Inc. The Principal Investigator ensures that all other persons involved in this project will maintain confidentiality as well.

Confirmation of the Principal Investigator:

Herewith I/we confirm to have understood and accepted all elements of the study protocol and the experimental part as agreed upon.

______________________________ ______________________________
Location/Institution Date

______________________________ ______________________________
Signature of Principal Investigator (PI) Printed name of PI

LUMICELL CONFIDENTIAL

This document is confidential. Do not disclose or use except as authorized.
