# Supplementary material for: Clinical Impact of Intraoperative Margin Assessment in Breast-Conserving Surgery With a Novel Pegulicianine Fluorescence–Guided System: A Nonrandomized Controlled Trial
Source: JAMA Surg. 2022 May 11;157(7):573–80. doi: 10.1001/jamasurg.2022.1075 (PMC9096689; doi:10.1001/jamasurg.2022.1075)
Supplement: Supplement 2. — Statistical analysis plan [file jamasurg-e221075-s002.pdf]

STATISTICAL ANALYSIS PLAN FOR LUMICELL PROTOCOL NUMBER CL0006r04

SAP Version Date: 05MAY2021

Study Title: FEASIBILITY STUDY PHASE C: EXPANSION INTO MULTIPLE INSTITUTIONS FOR TRAINING IN THE USE OF THE LUM IMAGING SYSTEM FOR INTRAOPERATIVE DETECTION OF RESIDUAL CANCER IN THE TUMOR BED OF FEMALE SUBJECTS WITH BREAST CANCER

System: LUM Imaging System (LUM015 imaging agent and LUM Imaging Device)

Authors:

Jorge Ferrer, Ph.D.
Senior VP, Clinical Research and Strategy
Lumicell, Inc.
jmferrer@lumicell.com
Phone: 617-571-0592

Manna Chang, Ph.D.
Principal Data Scientist
Lumicell, Inc.
mchang@lumicell.com

Gheorghe Doros, Ph.D.
Professor of Biostatistics
Department of Biostatistics
Boston University
doros@bu.edu

LUMICELL CONFIDENTIAL

This document is confidential. Do not disclose or use except as authorized.

TABLE OF CONTENTS

1 Introduction **2**

2 Analysis Populations **2**

3 Efficacy metrics analysis **2**

3.1 Definition of positive margins *2*

3.2 Removal of residual cancer (co-primary endpoint in pivotal study) *2*

3.3 Diagnostic performance analysis (co-primary endpoint in pivotal study) *3*

3.4 Other analyses (secondary endpoints in pivotal study) *5*

3.5 Safety measurements analysis *6*

3.6 Exploratory measurements *6*

4 Statistical Methods **6**

4.1 Demographic and baseline characteristics *6*

4.2 Residual cancer removal *7*

4.3 Diagnostic performance (sensitivity, specificity and Youden Index) *7*

4.4 Methods for additional analyses *7*

4.5 Safety analysis *7*

4.6 Protocol Deviations *8*

4.7 Reported device issues *8*

4.8 General considerations *8*

4.9 Exploratory data *11*

5 References **11**

This document is confidential. Do not disclose or use except as authorized.

1 Introduction

This document describes the statistical analysis plan to be performed for the Feasibility Study Phase C in breast cancer patients of the Lumicell Imaging System under protocol number CL0006r04. The objectives of this study are:

- Collect data to refine and verify the tumor detection algorithm
- Complete hands-on training of the surgeons and clinical staff that will be participating in the pivotal study to evaluate the efficacy and safety of the LUM Imaging System in breast cancer surgeries
- Identify and address any site-specific or user-specific issues for using the LUM Imaging System in breast cancer surgeries
- Collect safety and efficacy data

Although no pre-defined, hypothesis tested endpoints were included in the protocol, we will perform data analyses based on the proposed endpoint of the pivotal study protocol CL0007r07.

2 Analysis Populations

- Safety (SAF) population: the safety population includes all subjects who received study drug.
- Modified Intention To Treat (mITT) population: includes all SAF subjects excluding subjects who are not able to be imaged with the LUM Imaging System. That is, the mITT population includes all patients imaged with the LUM Imaging System and will be used as the primary analysis population for the efficacy metrics outlined in section 3.
- Per-Protocol Population: Includes mITT subjects who complete all study evaluations and are without any major protocol deviations. This per protocol population will be used to evaluate the efficacy metrics outlined in section 3.

3 Efficacy metrics analysis

3.1 Definition of positive margins

For this study, positive margins were defined using the latest consensus from the Society of Surgical Oncology as follows:

- For invasive cancer with or without associated carcinoma in situ: cancer cells present on ink [1]
- For pure DCIS lesions: DCIS present within 2 mm from the inked surface [2]. Note: when microinvasive cancer is reported, the positive margin criterion will be the same as for pure DCIS instead of the criterion for invasive cancer per NCCN Clinical Practice Guidelines in Oncology.

3.2 Removal of residual cancer (co-primary endpoint in pivotal study)

The first efficacy metric assesses how much additional residual is identified and removed that would have been missed during the initial standard of care procedure. This metric is defined as the ratio of patients who have **residual cancer** found in at least one LUM-guided shave (also known as therapeutic shave or "T-shave") among all patients. **Residual cancer** is defined as tumor found

This document is confidential. Do not disclose or use except as authorized.

by pathology in a therapeutic shave after the SOC surgical procedure is completed; that is, tumor that current SOC surgery failed to remove (Figure 1). Mathematically, it is defined as:

$$\frac{\#\ patients\ with\ residual\ cancer\ found\ in\ at\ least\ one\ therapeutic\ shave}{Total\ number\ of\ patients}$$

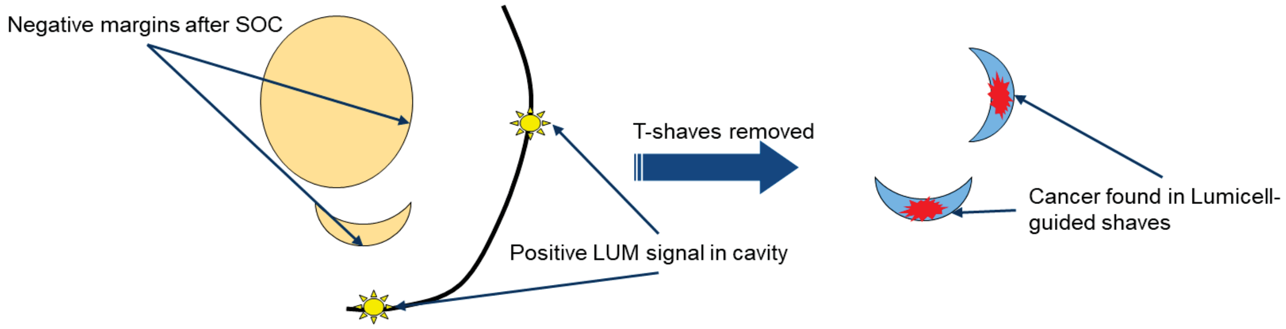

Figure 1: Depiction of removal of residual cancer analysis.

This proposed primary endpoint for determination of whether tumor is found in at least one therapeutic shave, the **ground truth** of histopathology assessment of the therapeutic shave is used

3.3 Diagnostic performance analysis (co-primary endpoint in pivotal study)

3.3.1 Sensitivity, specificity and Youden Index

The second efficacy metrics address the diagnostic accuracy of the system in identifying residual cancer, represented by sensitivity, specificity and Youden Index. The sensitivity, specificity and Youden Index (sensitivity + specificity -1) will be evaluated for the LUM Imaging System after SOC using the hierarchical approach for truth standard as defined below. This analysis also includes the lumpectomy margin sensitivity and specificity for predicting cancer remaining in the cavity as a comparison.

3.3.2 Definition of truth standard for diagnostic performance

The instrument diagnostic accuracy is measured by sensitivity and specificity on a per-tissue basis. Because a LUM-guided shave does not exist when a negative LUM image is indicated we propose a hierarchical approach to determine the truth standard as depicted in Figure 2. The highest truth standard is whether pathology finds cancer in a tissue shave removed from the area that was imaged in the cavity. When a shave does not exist, then whether tumor is found in a re-excision surgery is considered as the truth standard. When neither a shave nor a second surgery exists for a given orientation that was imaged, the margin assessment of that orientation is used as truth standard. We believe that the hierarchical approach outlined to determine the truth standard is the most meaningful and practical assessment of device accuracy to predict the presence of cancer in the cavity.

LUMICELL CONFIDENTIAL

This document is confidential. Do not disclose or use except as authorized.

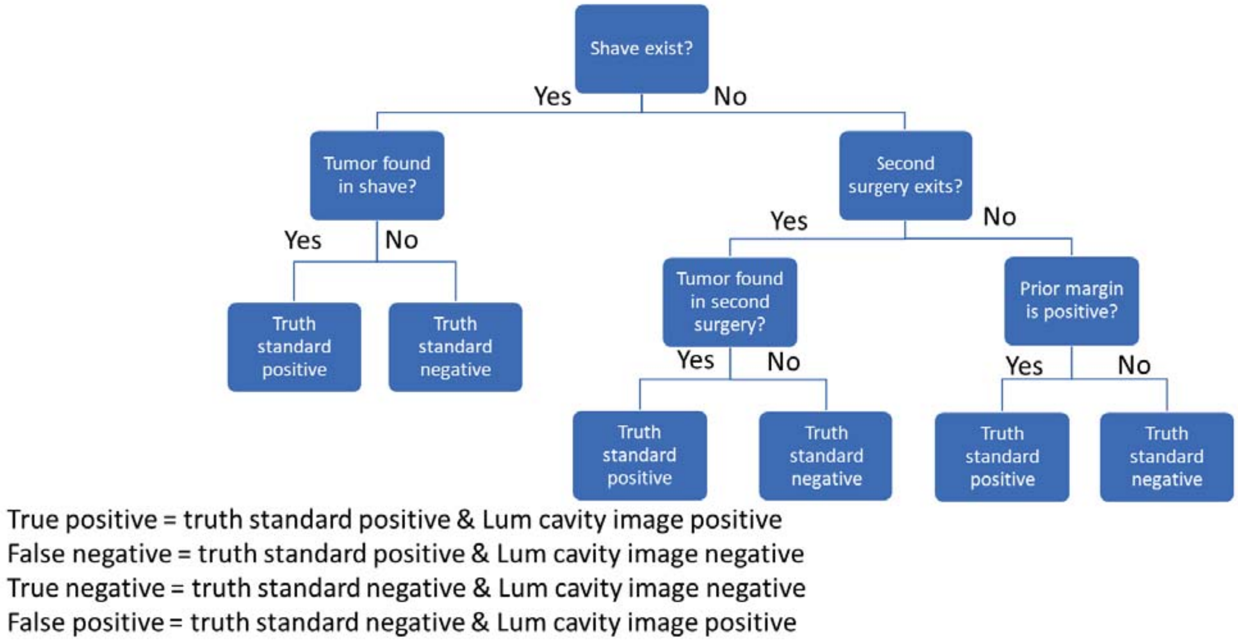

Figure 2: Definition of truth standard to evaluate the LUM Imaging System diagnostic performance.

When a therapeutic shave and re-excision are not available for an orientation, the margin from the prior resected tissue of the corresponding orientation will be used as ground inferred truth for evaluation (Figure 3).

The orientation of the tissue removed during a re-excision is typically noted in the pathology report and matches the orientation in which a positive margin was identified from the initial surgery. However, there will be instances in which the orientation of the tissue removed during a re-excision is not specified. In cases of unclear orientation of the tissue removed during re-excisions, it will be assumed that the re-excision was intended to address only the orientations with positive margins and pathology findings form the re-excision (tumor found/not fount) will be applied to those orientations.

If a mastectomy is performed as a follow-up surgery and no cancer is found in the mastectomy specimen, all the orientation will be considered as cancer negative for truth standard. If a mastectomy is performed and cancer is found but there is no indication on the orieantion in which the cancer was found, the orientation in which a positive margin was identified in the initial lumpectomy will be considered as cancer positive for truth standard unless otherwise noted.

Table 1: Definitions for 2x2 contingency table.

	Truth standard (+)	Truth standard (–)
LUM positive cavity signal (+)	True Positives	False Positives
LUM negative cavity signal (-)	False Negatives	True Negatives

LUMICELL CONFIDENTIAL

This document is confidential. Do not disclose or use except as authorized.

$$sensitivity = \frac{True\ Positives}{True\ Positives + False\ Negatives}$$

$$specificity = \frac{True\ Negatives}{True\ Negatives + False\ Positives}$$

$$Youden\ Index = sensitivity + specificity - 1$$

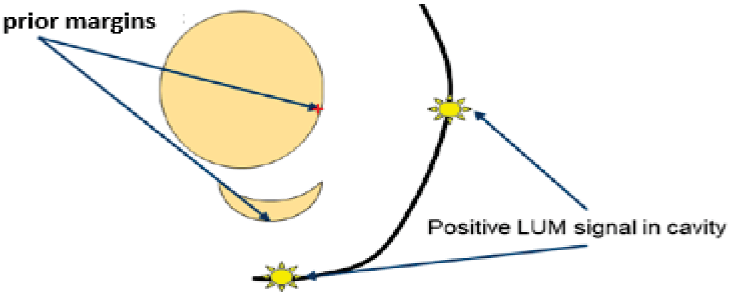

Figure 3: Demonstration of the prior margin for the LUM signal in the cavity,

3.4 Other analyses (secondary endpoints in pivotal study)

This endpoint is defined as:

a. Proportion of patients with positive-margins after standard of care breast-conserving surgery who have a LUM Imaging System signal in the cavity above the threshold as defined by the tumor detection algorithm (i.e., a positive LUM signal). The endpoint is estimated as:

$$\frac{\#\ positive\ margin\ patients\ after\ SOC\ with\ LUM\ signal\ above\ the\ threshold}{\#positive\ margin\ patients\ after\ SOC}$$

a. Proportion of subjects with pathology-positive margins after standard of care breast-conserving surgery, for whom additional therapeutic shaves resulted in pathology-negative margins. Note: This efficacy metric also estimates the potential reduction in re-excision surgeries following standard of care breast-conserving surgery that resulted from a positive SOC margin. This is estimated as:

$$\frac{\#\ positive\ margin\ patients\ after\ SOC\ with\ all\ final\ negative\ margins\ after\ the\ LUM\ procedure}{\#positive\ margin\ patients\ after\ SOC}$$

b. Mean absolute volume (cc) of tissue removed from therapeutic shaves. For each subject, absolute shave volume will be calculated as length (c) × width (c) × depth (c) for each therapeutic shave with the sum by subject of all therapeutic shaves. Dimensions for the tissue will be obtained as entered in the case report form from the standard of care pathology report. Subjects with no therapeutic shaves taken will have a therapeutic shave volume of 0cc. The proportion of the subjects with and without therapeutic shaves will be reported. The mean and median (with 90% confidence interval) of the total surgery volume of tissue excised and total volume of the SOC tissue will be reported grouped by the subjects with or without therapeutic shaves.

LUMICELL CONFIDENTIAL

This document is confidential. Do not disclose or use except as authorized.

c. Mean contribution of therapeutic shave volume to total volume of tissue removed. The ratio of therapeutic shave volume to total volume of resection (main specimen + SOC shave volume + therapeutic shave volume) tissue removed will be calculated in the total population and in the population with therapeutic shaves removed. For each subject, the volume for each piece of tissue will be calculated as length (c) × width (c) × depth (c), and the dimensions will be obtained as entered in the caser report form from the standard of care pathology report. The mean and median (with 90% confidence interval) will be reported.
d. Average number of SOC shaves and therapeutic shaves taken per subject overall and by type of SOC lumpectomy procedure (i.e., lumpectomy with comprehensive shaves vs. lumpectomy with or without selective shaves).
e. Number of second surgeries recommended as a result of final positive margins.
f. Rate of second surgery procedures as a result of final positive margins.
g. Rate of second surgery procedures as a result of final positive margins.
h. Rate of cancer found in second surgeries.
i. Number of second surgeries for each subject.
j. Number of images per subject from the cavity after SOC and first round of the therapeutic shaves.
k. Number of LUM images having LUM-positive signal and therapeutic shaves taken.
l. Fluorescence intensity mean of the cavity images, LUM positive images and positive images with shave after SOC and after one round of the therapeutic shaves.
m. Number of device issues and malfunctions and their impact to data capture.

3.5 Safety measurements analysis

The safety measurements include the following:

a. Adverse events stratified by severity and relatedness to drug/device
b. Serious adverse events stratified by severity and relatedness to drug/device
c. Adverse events by preferred term (sorted by descending occurrences for each preferred term)
d. Summary of adverse events, overall and split by expectedness to drug/device
e. Comparison of adverse events in subjects that have at least one therapeutic shave versus subjects that have no therapeutic shaves

3.6 Exploratory measurements

- Collect exploratory data on tissue types found in therapeutic shaves

4 Statistical Methods

4.1 Demographic and baseline characteristics

Demographic and baseline characteristic summaries will be provided. These measures include; age, race and ethnicity, menopausal status, mammographic breast density, palpability, cancer histologic type, tumor size, node positive patients, and tumor receptor status (ER/PR/HER2), and baseline body mass index (BMI) calculated from weight and height. Statistics for continuous variables will include mean, median, standard deviation (SD), minimum, maximum and sample

This document is confidential. Do not disclose or use except as authorized.

size for the overall sample and for subgroups. Binary variables will be described with frequencies and percentages for the overall sample and subgroups. Demographic and baseline characteristics will be summarized by overall descriptive statistics for analysis populations

4.2 Residual cancer removal

This proportion will be evaluated using Binomial distribution method to report the point value and the two-sided 95% confidence interval.

4.3 Diagnostic performance (sensitivity, specificity and Youden Index)

Sensitivity and specificity will be reported based on the 2x2 contingency table (Table 1) including the point value as well as the two-sided 95% confidence interval.

Youden Index will be evaluated using the relationship:

$$Youden\ Index = sensitivity + specificity - 1 = sensitivity - false\ positive\ rate$$

where

$$false\ positive\ rate = 1 - sensitivity = \frac{False\ Positives}{True\ Negatives + False\ Positives}$$

A difference of proportions approach will be used to report the Youden Index point value and the two-sided 95% confidence interval.

4.4 Methods for additional analyses

Secondary outcomes involving proportions will be analyzed using methods for Binomial distributions akin to the ones described above. Continuous measurement will be summarized using means and standard deviation. The percent of people with a specific AE will be reported in prespecified groups. The percent of subjects with adverse events in subjects that have at least one therapeutic shave will be compared with the percent of subjects with adverse events that have no therapeutic shaves using Fisher's exact test.

4.5 Safety analysis

Safety data will be reported as:

- Adverse events stratified by severity and relatedness to drug/device.
- Serious adverse events stratified by severity and relatedness to drug/device.
- Adverse events coded per MedDRA guidelines
- Adverse events by preferred term per MedDRA (sorted by descending occurrences for each preferred term).

Adverse events will be reported by the clinical trial sites and data is recorded in the eCRF. However, a Lumicell representative is responsible for assigning the 'expectedness' of any event as described in the study plans or protocols

4.5.1 Complement activation data

This document is confidential. Do not disclose or use except as authorized.

Raw data will be generated by Lab Connect for blood samples collected in response to an adverse event or as part of the sub-study. These raw data sets are data external to the eCRF.

4.6 Protocol Deviations

Protocol Deviations are identified by either Lumicell personnel or clinical sites. Deviations are reported in the eCRF. Lumicell personnel will adjudicate these events as described in the study plans and procedures. The adjudication will include an impact to data capture and may be removed from the efficacy analysis. The Protocol Deviations external data set will be used for analysis and not generated by the protocol deviations reported in the eCRF.

4.7 Reported device issues

Device issues as reported or interpreted by the clinical site are captured in the eCRF. These reported device issues will be evaluated and adjudicated by Lumicell personnel for impact to subject safety, impact to data capture, and categorization. Issues that resulted in an impact to data capture may be removed from the efficacy analysis. The 'Device Issues' external data set will be used for analysis and not generated by the device issues reported in the eCRF.

4.8 General considerations

All analyses will be performed under Good Clinical Practice (GCP) standards using the statistical analysis plan establish in this document. All analyses will be conducted, and tables and listings generated using Statistical Analysis System (SAS®) Version 9.4 or higher. Graphics may be prepared with SAS Version 9.4 or higher; R version 3.6.1 or higher (R Core Team (2019)), SigmaPlot 12.3 or higher (Systat Software, Inc., San Jose, California). Subject level data will be provided as a listing to support tables and figures. Summaries will be provided overall, by device size, and by site.

4.8.1 Numerical precision

For reporting of descriptive statistics, the mean, SD, median, min, max and quantile will be presented to 1-digit precision. The minimum, median, and maximum will be presented to the same precision as the source data. The maximum number of decimals will be two, no matter how precise the source data is. The percentages will be reported to 1 decimal (format of xx.x%). Sensitivity and specificity will be reported as percentages with 1-digit precision (format of xx.x%). P-values will be reported to three (3) decimal places or as < 0.001.

4.8.2 Multiple images to cover entire shave or multiple shaves for one image

To accurately evaluate the study endpoints, it is critical that images are collected from the entire lumpectomy cavity surface. To achieve this, multiple images may be required to record the LUM assessment for an entire orientation (e.g. 2 images to cover the entire superior orientation) but a single shave may exist. When multiple images exist for a given orientation or shave, the combination of the images will be considered as one single output as described below:

- All images for a given orientation or shaved region are LUM negative, then the LUM result for the entire orientation or shaved region is considered as LUM negative.

This document is confidential. Do not disclose or use except as authorized.

- If one or more of the images for a given orientation or shaved region is LUM positive, then the LUM result for the entire orientation or shaved region is considered as LUM positive.

When there are more than one shave for a single image, the pathology finding for the combination of the shaves will be considered as one as follows:

- If no tumor is found on the shaves, then the combination of the shaves is considered as truth standard negative.
  If any of the shaves has tumor, then the combination of the shaves is considered as truth standard positive.

4.8.3 Missing and spurious data

During Lumicell-guided imaging, when there is not enough tissue to be removed (e.g. too close to skin or too close to the chest wall) no LUM image shall be recorded because it does not generate an actionable result.

In the case where the LUM Imaging System indicates that a therapeutic shave needs to be removed but the surgeon decides not to follow this guidance, the reason for not doing so will be recorded in the patient's CRF or during imaging. These instances are considered as a protocol deviation. There are other cases when the shavings reach protocol limits so no shaving will be performed. In these cases, the data will be included in the performance evaluation as is - that is no Lumicell-guided shave-taken will be considered as no removing of cancer or positive margin. However, they will be considered as a detection if there are cancer in the re-excision or if there are positive margins on the corresponding orientations.

Occasionally, a cavity image may be recorded from an unintended region or the image may be deemed as low quality (blurry, out of focus, etc.). For example, the surgeon realizes that he/she placed the device in the wrong location within the cavity after recording an image. Since the LUM software does not allow to overwrite saved images, this situation will be documented, the image will be labeled as "image retaken", "eval", "initialscan" or "transient" and the image will be excluded from the analysis. In addition, images labeled as "lens issue" will be considered as a device malfunction and will be excluded from the analysis.

Other cases are handled as follows:

a. When a patient was prescribed Letrozole adjuvant therapy prior to surgery, the patient will be removed from the device performance evaluation.
b. When the time from injection of LUM015 to lumpectomy incision is greater than 6 hours due to unplanned surgical delays, the patients will be removed from the device performance evaluation.
c. For the re-excision rate endpoints, all patients undergoing a re-excision will be included in the analysis.
d. When an image is indicated negative by LUM Imaging System but the shave is taken due to X-ray or pathology suggestion, it will be kept for sensitivity and specificity evaluation, however, for the analysis related to margins, it is conditional and described in g.

LUMICELL CONFIDENTIAL

This document is confidential. Do not disclose or use except as authorized.

e. In the case of a therapeutic shave taken but no image saved before the shave was removed, the shave will be excluded from analysis.
f. In the case of the subject with minor deviation, like no blood drawn, high blood pressure, etc., data will be kept for device performance evaluation.
g. In the case of SOC shave is taken after use of the Lumicell imaging system, if there is an image for this shave, it will be kept for sensitivity and specificity analysis. If there is a therapeutic shave ahead of the SOC shave but the orientation of the SOC shave is not the same as that of the therapeutic shave taken before it, this SOC shave will be kept for the margin analysis. If the orientation of the SOC shave is same as that of the therapeutic shave taken before it, this SOC shave should be removed from the margin analysis. If there is no therapeutic shave ahead of the SOC shave, this deviated SOC shave will be kept as SOC shave for margin analysis.
h. In the case of a patient having bilateral lumpectomy, only the data collected from the trial side will be included in the device performance evaluation.
i. In the case of multiple reports margins and tumors for one block of shave, the distance of the closest margin will be used and 'Yes' for the shave containing tumor if at least one report for that block is 'Yes'.
j. In the case of the unknown dimension of the shaves or 999 for any of the dimensions, the volume of the shave is the average of that of all the existing shaves of the corresponding type (SOC or Therapeutic).
k. In the case of a tissue has unknown or unreported distances to margins, the data in the eCRF will indicate "unknown", it will be interpreted as no specified distance has been reported or the options in the eCRF do not fit the distance description in the pathology report and/or the pathology worksheet. However, the determination for positive or negative margins in the corresponding orientations will depend the pathology determination on whether margins are positive or negative. For example, a pathology report may state "closest margin to invasive cancer is 1mm in medial". Thus it is assumed that the margins for the other orientaions are > 1mm but the specific distance is not reported. For orientations other than medial, the eCRF may indicate "unknown" for margin distance, but the margin status will be considered as "negative" for data analysis.
l. For reported microinvasive cancer, adjudicated correction will be provided external to the eCRF and will be used to change the margin type from Invasive to DCIS following NCCN guidalines.
m. Margins for SOC and final margins will be derived from the tumor distance to margins. If there is a discrepancy between the derived margins based on distance and the margin status determined by the clinical site (could be due to different criteria used by the clinical sites), adjudication of the margin status will be done by Lumicell based protocol defined margin criteria (section 3.1).

4.8.4 Not enough tissue left for imaging or removing a therapeutic shave

This document is confidential. Do not disclose or use except as authorized.

This is a list of certain orientations in a given subject in which there is not enough tissue left for further imaging or removing a therapeutic shave. The margins of these orientations in the list will be ignored and will be treated as negative margin from the efficacy analyses.

4.9 Exploratory data

Exploratory analysis of the types of tissue found in the therapeutic shaves is based on "free text" entered in the eCRF.

5 References

1. Moran, M.S., et al., *Society of Surgical Oncology-American Society for Radiation Oncology consensus guideline on margins for breast-conserving surgery with whole-breast irradiation in stages I and II invasive breast cancer.* Int J Radiat Oncol Biol Phys, 2014. **88**(3): p. 553-64.
2. Morrow, M., et al., *Society of Surgical Oncology-American Society for Radiation Oncology-American Society of Clinical Oncology Consensus Guideline on Margins for Breast-Conserving Surgery With Whole-Breast Irradiation in Ductal Carcinoma In Situ.* J Clin Oncol, 2016.

LUMICELL CONFIDENTIAL

This document is confidential. Do not disclose or use except as authorized.
