# Supplementary material for: Clinical Impact of Intraoperative Margin Assessment in Breast-Conserving Surgery With a Novel Pegulicianine Fluorescence–Guided System: A Nonrandomized Controlled Trial
Source: JAMA Surg. 2022 May 11;157(7):573–80. doi: 10.1001/jamasurg.2022.1075 (PMC9096689; doi:10.1001/jamasurg.2022.1075)
Supplement: Supplement 3. — eFigure 1. Definition of truth standards used to evaluate pFGS image result or SOC margin assessment at identifying tumor in the corresponding cavity orientation eFigure 2. pFGS Outcomes Flowchart eFigure 3. Final margin status of patients with positive margins following SOC excision, candidates for re-excision procedure (n=38) eTable 1. Participating site location and enrollment eTable 2. Tissue Type Distribution in pFGS-guided shaves eTable 3. Diagnostic accuracy of the pFGS system for predicting residual cancer in the lumpectomy cavity: patient level analysis (n=230 patients) eTable 4. Excisional volumes stratified by surgical technique [file jamasurg-e221075-s003.pdf]

Supplemental Online Content

Hwang ES, Beitsch P, Blumencranz P, et al; INSITE study team. Clinical impact of intraoperative margin assessment in breast-conserving surgery with a novel pegulicianine fluorescence–guided system: a nonrandomized controlled trial. *JAMA Surg*. Published online May 11, 2022. doi:10.1001/jamasurg.2022.1075

eFigure 1. Definition of truth standards used to evaluate pFGS image result or SOC margin assessment at identifying tumor in the corresponding cavity orientation

eFigure 2. pFGS Outcomes Flowchart

eFigure 3. Final margin status of patients with positive margins following SOC excision, candidates for re-excision procedure (n=38)

eTable 1. Participating site location and enrollment

eTable 2. Tissue Type Distribution in pFGS-guided shaves

eTable 3. Diagnostic accuracy of the pFGS system for predicting residual cancer in the lumpectomy cavity: patient level analysis (n=230 patients)

eTable 4. Excisional volumes stratified by surgical technique

This supplemental material has been provided by the authors to give readers additional information about their work.

eFigure 1. Definition of truth standards used to evaluate pFGS image result or SOC margin assessment at identifying tumor in the corresponding cavity orientation

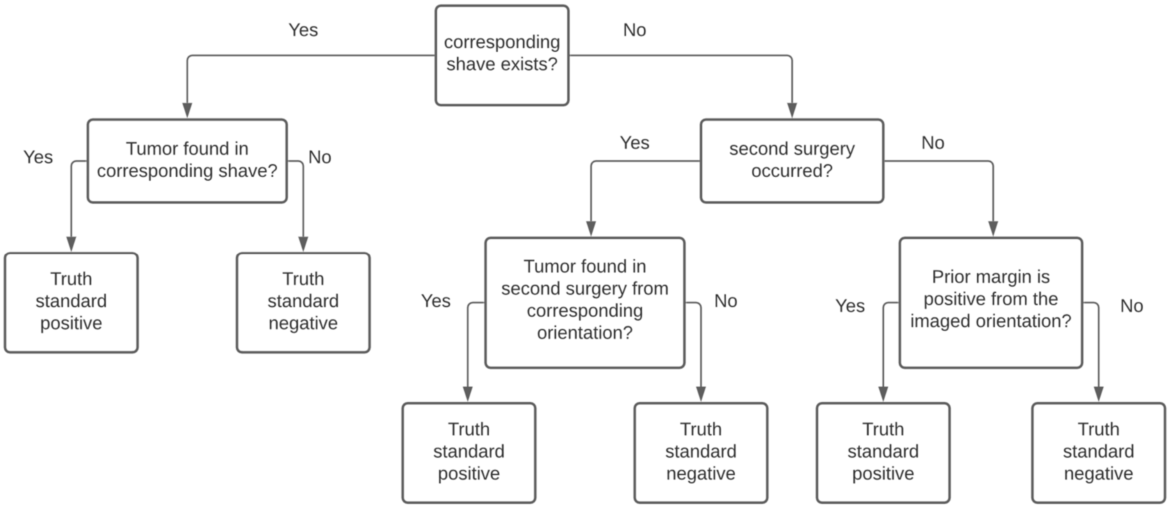

True positive = truth standard positive & pFGS image/SOC margin positive.
False negative = truth standard positive & pFGS image/SOC margin negative.
True negative = truth standard negative and pFGS image/SOC margin negative.
False positive = Truth standard negative and pFGS image/SOC margin positive.

© 2022 American Medical Association. All rights reserved.

eFigure 2. pFGS Outcomes Flowchart

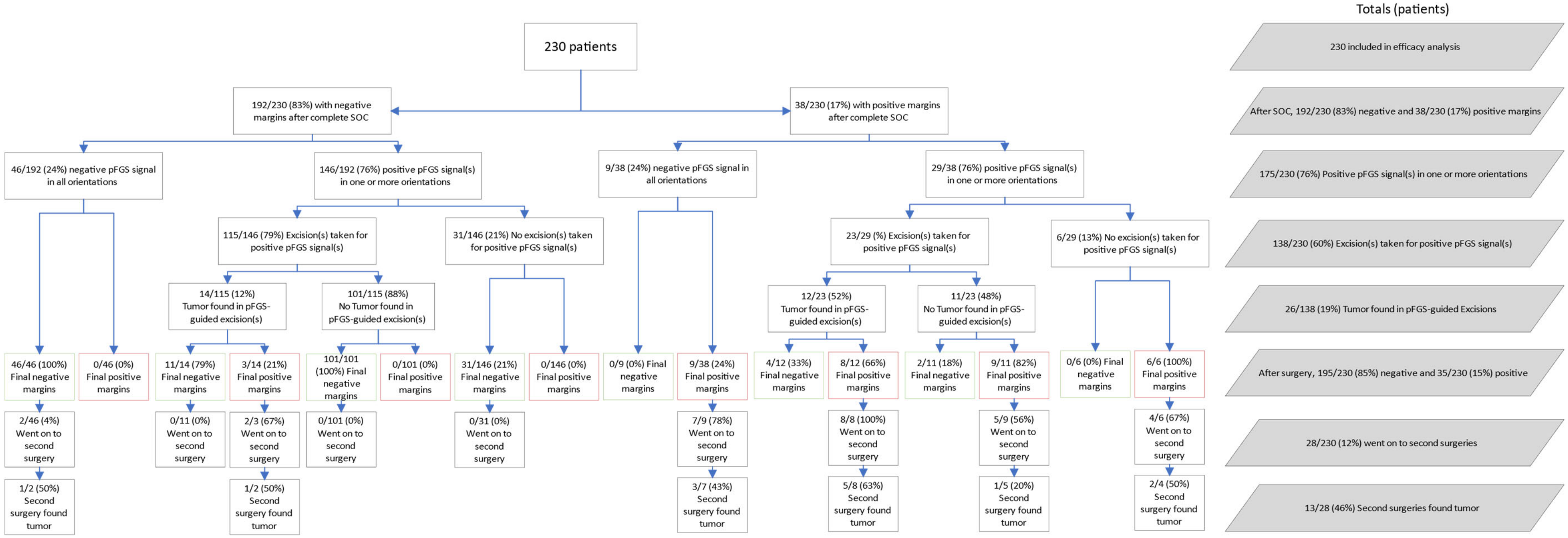

eFigure 3. Final margin status of patients with positive margins following SOC excision, candidates for re-excision procedure (n=38)

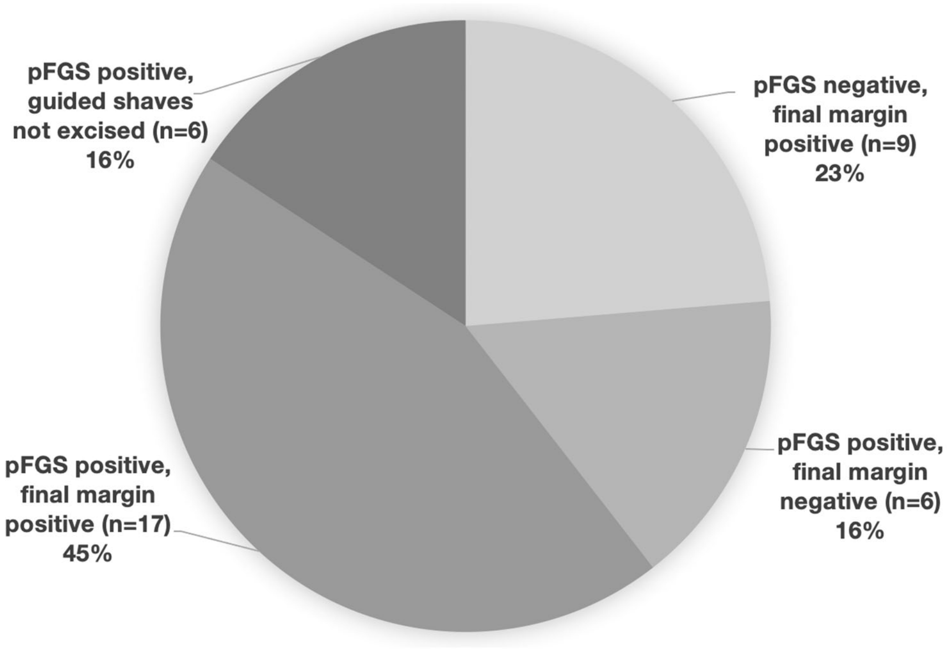

eTable 1. Participating site location and enrollment

Site	Location	Patients Enrolled (n=230), n (%)
Novant Health	Winston-Salem, North Carolina	38 (16.5)
Massachusetts General Hospital	Boston, Massachusetts	36 (15.6)
Comprehensive Breast Care Center	Clearwater, Florida	22 (9.6)
Penn State Hershey Medical Center	Hershey, Pennsylvania	15 (6.5)
MD Anderson Cancer Center	Houston, Texas	14 (5.7)
University of South Alabama	Mobile, Alabama	13 (5.7)
Beaumont Royal Oak	Royal Oak, Michigan	13 (5.7)
Stanford University Medical Center	Palo Alto, California	11 (4.8)
Baptist MD Anderson Cancer Physicians	Jacksonville, Florida	11 (4.8)
Beaumont Troy	Troy, Michigan	11 (4.8)
Duke University Medical Center	Durham, North Carolina	9 (3.9)
Cleveland Clinic	Cleveland, Ohio	9 (3.9)
Franciscan Breast Surgery	Tacoma, Washington	9 (3.9)
Yale University School of Medicine	New Haven, Connecticut	8 (3.5)
Dallas Breast Center	Dallas, Texas	8 (3.5)
Lahey Hospital and Medical Center	Burlington, Massachusetts	3 (1.3)

© 2022 American Medical Association. All rights reserved.

eTable 2. Tissue Type Distribution in pFGS-guided shaves

	**pFGS-guided shaves (n=243), n (%)**	
**Tissue Type**	**Cancer identified (n=34)**	**No cancer identified (n=209)**
**Cancer Type**		
Invasive	10 (29.4)	NA
DCIS only	24 (70.6)	NA
**Benign Tissue Type**		
Inflammation	1 (2.9)	38 (18.2)
Atypical ductal hyperplasia	NA	4 (1.9)
Usual ductal hyperplasia	2 (5.9)	20 (9.6)
Atypical lobular hyperplasia	NA	6 (2.9)
LCIS	2 (5.9)	4 (1.9)
Fibroadenoma	1 (2.9)	3 (1.4)
Papilloma	NA	3 (1.4)
Columnar cell change	NA	2 (1.0)
Apocrine metaplasia	2 (5.9)	5 (2.4)
Adenosis	NA	10 (4.8)
Biopsy site change	2 (5.9)	4 (1.9)
Radial scar	3 (8.8)	4 (1.9)
Fibrocystic change	2 (5.9)	24 (11.5)
Dense fibrous	2 (5.9)	28 (13.4)
Benign breast tissue only	NA	100 (47.8)
Unknown	NA	8 (3.8)

© 2022 American Medical Association. All rights reserved.

eTable 3. Diagnostic accuracy of the pFGS system for predicting residual cancer in the lumpectomy cavity: patient level analysis (n=230 patients)

**N=230**	**True Margin Status (based on SOC margin status)[a]**	
Predicted Margin Status (positive/negative pFGS imaging)	True positives (n=29)	False positives (n=146)
	False negatives (n=9)	True negatives (n=46)
False negative rate	9/38	23.7%
False positive rate	146/192	76.0%
Positive predictive value	29/175	16.6%
Negative predictive value	46/55	83.6%
Sensitivity	29/38	76.3%
Specificity	46/192	24.0%

[a] TN: negative margins after SOC excision, negative pFGS signal; TP: positive margins after SOC excision, positive pFGS signal; FN: positive margins after SOC excision, negative pFGS signal; FP: negative margins after SOC excision, positive pFGS signal.

© 2022 American Medical Association. All rights reserved.

Supplemental Table 4. Excisional volumes stratified by surgical technique.

Excision volume	Patients that completed the pFGS imaging procedure			
	All (n=230)	Comprehensive SOC Shaves[a] (n=58)	Selective SOC Shaves[b] (n=86)	No SOC Shaves[c] (n=86)
Lumpectomy volume, median (2.5%, 97.5%), cm^3	57.8 (11.5, 252.7)	35.7 (7.6, 186.3)	60.0 (13.8, 193.1)	66.1 (22.6, 276.9)
SOC shave volume, median (2.5%, 97.5%), cm^3	6.0 (0.0, 52.5)	15.4 (5.0, 52.5)	10.2 (1.1, 63.0)	NA
SOC total volume, median (2.5%, 97.5%), cm^3	65.4 (17.9, 276.9)	54.1 (14.1, 234.1)	73.2 (17.9, 267.5)	66.1 (22.6, 276.9)
pFGS shave volume, median (2.5%, 97.5%), cm^3	4.0 (0.0, 102.8)	1.4 (0.0, 27.0)	3.9 (0.0, 125.4)	10.0 (0.0, 91.3)
Total volume, median (2.5%, 97.5%), cm^3	77.7 (18.8, 336.8)	61.6 (14.1, 234.1)	91.0 (21.1, 342.4)	82.2 (23.6, 336.8)
Number of pFGS shaves, mean (SD)	1.1 (1.2)	1.0 (1.1)	1.0 (1.0)	1.2 (1.4)

[a] Comprehensive SOC shaves: Additional tissue removed from all surfaces of the cavity walls.

[b] Selective SOC shaves: Additional tissue removed from specific locations in the cavity walls after resection of the main specimen based on intraoperative imaging, palpation, pathology, etc.

[c] No SOC shaves: No additional tissue removed from the cavity walls after resection of the main specimen.

© 2022 American Medical Association. All rights reserved.
