# Supplementary material for: Clinical Impact of Intraoperative Margin Assessment in Breast-Conserving Surgery With a Novel Pegulicianine Fluorescence–Guided System: A Nonrandomized Controlled Trial
Source: JAMA Surg. 2022 May 11;157(7):573–80. doi: 10.1001/jamasurg.2022.1075 (PMC9096689; doi:10.1001/jamasurg.2022.1075)
Supplement: Supplement 4. — The INSITE study team [file jamasurg-e221075-s004.pdf]

Supplemental Online Content: Nonauthor Collaborators

*Indicates required information. Only first name, last name, and suffix will appear in PubMed.

***Group Name(s): INSITE Study Team**							
***First Name and Middle Initial(s)**	***Last Name**	***Suffix (eg, Jr, III)**	Academic Degrees	Institution	Location (city, state/province, country)	Role or Contribution, eg, chair, principal investigator	Group (if more than 1 Group listed in the byline) and/or Subgroup (eg, Steering Committee)
Jorge	Ferrer		PhD	Lumicell, Inc.	Newton, MA, USA	sponsor	INSITE Study Team
Brian	Schlossberg		PhD	Lumicell, Inc.	Newton, MA, USA	sponsor	INSITE Study Team
Kate	Smith		MPH	Lumicell, Inc.	Newton, MA, USA	sponsor	INSITE Study Team
Daniel K	Harris		PhD	Lumicell, Inc.	Newton, MA, USA	sponsor	INSITE Study Team
David	Strasfeld		PhD	Lumicell, Inc.	Newton, MA, USA	sponsor	INSITE Study Team
David	Lee		MS	Lumicell, Inc.	Newton, MA, USA	sponsor	INSITE Study Team
Manna	Chang		PhD	Lumicell, Inc.	Newton, MA, USA	sponsor	INSITE Study Team
Sean	Madden		PhD	Lumicell, Inc.	Newton, MA, USA	sponsor	INSITE Study Team
