# Supplementary material for: Clinical Impact of Intraoperative Margin Assessment in Breast-Conserving Surgery With a Novel Pegulicianine Fluorescence–Guided System: A Nonrandomized Controlled Trial
Source: JAMA Surg. 2022 May 11;157(7):573–80. doi: 10.1001/jamasurg.2022.1075 (PMC9096689; doi:10.1001/jamasurg.2022.1075)
Supplement: Supplement 5. — Data sharing statement [file jamasurg-e221075-s005.pdf]

Data Sharing Statement

Hwang. Clinical Impact of Intraoperative Margin Assessment in Breast Cancer Surgery With a Novel Pegulicianine Fluorescence-Guided System. *JAMA Surg*. Published May 11, 2022.
doi:10.1001/jamasurg.2022.1075

Data
Data available: Yes
Data types: Deidentified participant data
How to access data: shelley.hwang@duke.edu
When available: With publication

Supporting Documents
Document types: None

Additional Information
Who can access the data: researchers whose proposed use of the data has been approved by the INSITE Study Team
Types of analyses: any purpose
Mechanisms of data availability: after approval of a proposal with signed DTA and MTA
